# PRSice-2: Polygenic Risk Score software for biobank-scale data

**DOI:** 10.1093/gigascience/giz082

**Published:** 2019-07-15

**Authors:** Shing Wan Choi, Paul F O'Reilly

**Affiliations:** 1 MRC Social, Genetic and Developmental Psychiatry Centre, Institute of Psychiatry, Psychology and Neuroscience, King's College London, De Crespigny Park, Denmark Hill, London, UK, SE5 8AF; 2 Department of Genetics and Genomic Sciences, Icahn School of Medicine, Mount Sinai, 1 Gustave L. Levy Pl, New York City, NY 10029, USA

**Keywords:** polygenic risk score, GWAS, imputation

## Abstract

**Background:**

Polygenic risk score (PRS) analyses have become an integral part of biomedical research, exploited to gain insights into shared aetiology among traits, to control for genomic profile in experimental studies, and to strengthen causal inference, among a range of applications. Substantial efforts are now devoted to biobank projects to collect large genetic and phenotypic data, providing unprecedented opportunity for genetic discovery and applications. To process the large-scale data provided by such biobank resources, highly efficient and scalable methods and software are required.

**Results:**

Here we introduce PRSice-2, an efficient and scalable software program for automating and simplifying PRS analyses on large-scale data. PRSice-2 handles both genotyped and imputed data, provides empirical association *P*-values free from inflation due to overfitting, supports different inheritance models, and can evaluate multiple continuous and binary target traits simultaneously. We demonstrate that PRSice-2 is dramatically faster and more memory-efficient than PRSice-1 and alternative PRS software, LDpred and lassosum, while having comparable predictive power.

**Conclusion:**

PRSice-2's combination of efficiency and power will be increasingly important as data sizes grow and as the applications of PRS become more sophisticated, e.g., when incorporated into high-dimensional or gene set–based analyses. PRSice-2 is written in C++, with an R script for plotting, and is freely available for download from http://PRSice.info.

Polygenic risk score (PRS) analyses are beginning to play a critical role in biomedical research, being already sufficiently powered to provide scientific insights and with the potential to contribute to stratified medicine in the future [1–9]. The increasing availability of genetic data from regional and national biobank projects [10–12] has allowed more powerful PRSs to be calculated. However, the calculation of PRS, which involves parameter optimization [13–16], can be a computationally intensive process, especially for large datasets and when multiple analyses are conducted.

To fully utilize the power of large datasets and to facilitate future method and application developments, at scale, we have performed a major overhaul of our original PRSice software [13] to produce PRSice-2. All code has been rewritten in C++, and code from PLINK-1.9 [17] has been incorporated to optimize computation. As a result of the consistent language and switch to objected-oriented code, different analytical components of the code can communicate directly, without, e.g., the generation of intermediate files, such as those containing PRS corresponding to each *P-*value threshold, or post-processed genotype files. This has generated a substantial speed-up, a lower processing burden, and a reduction in disk space requirement in PRSice-2. In addition, a separate plotting script is implemented in R. Separate tasks are organized into functions and are, thus, more amenable to tailored extensions by users. Finally, a range of user options are incorporated into PRSice-2 to increase flexibility and improve usability.

## Features of PRSice-2

PRSice-2 uses the same standard approach to PRS calculation as PRSice, involving clumping single-nucleotide polymorphisms (SNPs) (thinning SNPs according to linkage disequilibrium and *P*-value) and then performing *P*-value thresholding, known as the “C+T” method [14], and retains the majority of the features of its predecessor [13], including automatic strand flipping, clumping [18], and calculation and evaluation of PRS under few (“fastscore”) or many (“high-resolution scoring”) *P*-value thresholds.

When compared to PRSice, PRSice-2 streamlines the entire PRS analysis pipeline without generating intermediate files, and performs all the main computations in C++, leading to a drastic speed-up in run time and reduction in memory burden (see Supplementary Fig. 1). Extraction and exclusion of samples and SNPs are also implemented, allowing PRS analysis to be performed directly on a subset of the input data without performing pre-filtering.

Briefly, the main features of PRSice-2 are:

1. Handles large-scale PRS analyses of both genotyped and imputed data
2. Computes empirical association *P-*values to account for overfitting
3. Can perform PRS analyses on a large number of target phenotypes simultaneously
4. Provides several options for imputing missing genotypes
5. Allows calculation of PRS based on different inheritance models, including additive, dominant, recessive, and heterozygous models
6. Automatically generates dummy variables for categorical covariates
7. Can perform regression to estimate relative effect/risk corresponding to samples in user-defined stratum of the population. Can output quantile and strata plots
8. Amenable to user extensions, such as relating to input data format, regression modelling, and output

## Handling of Imputed Data

Genotypes are typically represented as the discrete counts of the minor or effect allele (0, 1, or 2), for SNPs, in each individual. Genotypes not included in the genotyping chip can, potentially, be imputed and are usually either recorded as a set of 3 probabilities corresponding to the probability of each of the possible genotypes [19] or, based on these, as the expected genotype (a real number between 0 and 2 known as the “dosage”) [19] or as the “best-guess” (most probable) genotype. While any of these data formats can be exploited in PRS analyses, the most common approach is to use the best-guess genotype for each individual. However, this approach does not account for the uncertainty in the imputed genotype.

Currently, most PRS software only supports input of the genotyped format. Therefore, users need to generate a large intermediate file containing the best-guess genotypes and discard any information related to imputation uncertainty. To reduce the storage space requirement and to incorporate imputation uncertainty into PRS analyses, PRSice-2 implements support for the BGEN imputation format. PRSice-2 can directly process the BGEN imputed format and convert to either best-guess genotypes or dosages when calculating the PRS, without generating a large intermediate file. While PRSs based on best-guess genotypes are calculated as for genotyped input, dosage-based PRSs are calculated as

(1) \begin{equation*} \mathrm{PRS}\ = {\mathop \sum \nolimits_i^m {\beta _i}\left( {\mathop \sum \nolimits_{j = 0}^2 {\omega _{ij}} \times j} \right)}, \end{equation*}

where ${\omega _{ij}}$ is the probability of observing genotype *j*, where *j* ∈ {0,1,2}, for the *i* th SNP; *m* is the number of SNPs; and ${\beta _i}$ is the effect size of the *i*th SNP estimated from the relevant base genome-wide association study (GWAS) data.

The ability to perform PRS analyses directly on imputed data can be particularly useful when the base GWAS and target samples are genotyped on a different platform because then there can be a small fraction of overlapping SNPs. For example, of the 725,459 post–quality control SNPs (see Supplementary Note 1) in the UK Biobank genotype data [10], only 31% (222,956) were found in the GIANT Height and Body Mass Index (BMI) GWAS [20,21]. The use of imputed SNPs increases the number of overlapping SNPs to 2,121,036 SNPs. To assess the gain in power when using imputed vs un-imputed data, we performed PRS analyses on height and BMI using UK Biobank genotyped and imputed data, with GWAS summary statistics provided by the GIANT consortium [20,21]. Age, sex, UK Biobank genotyping batch, UK Biobank assessment centre, and 40 principal components were first regressed out from the phenotype and the standardized residuals were used instead.

We performed a linear regression using PRSice-2, with the UK Biobank data as target sample using the default parameters. When PRS is calculated from the best-guess genotype, the best-guess genotype is defined as the genotype having an imputation probability of ≥0.9. If there is no such genotype, then the SNP is considered to be missing for the individual. In addition, for the imputed data, we filtered out SNPs with imputation quality score <0.8. With height as the outcome and PRS for height as predictor, we observed an increase in phenotypic variance explained (*R*^2^) of the PRS from 0.145 when using genotyped data to 0.152 when using best-guess imputed genotypes, and 0.153 when using dosage data; likewise, the *R*^2^ for BMI increased from 0.0475 when using genotype data to 0.0529 when using best-guess genotypes, and to 0.0535 when using dosage data. These results exemplify the potential gain in predictive power when using dosage data compared to using genotyped or best-guess genotype data. However, given the modest increases in predictive power, users may wish to perform first-pass analyses on genotyped-only data before application to the more computationally intensive imputed data. A further challenge in exploiting imputed data is that there are numerous imputed formats in use in the field. While it is difficult to support all imputed formats, PRSice-2 adopts a modular approach, which allows simple incorporation of supports for additional data formats (e.g., VCF) in the future.

## Calculation of Empirical *P*-value

All approaches to PRS calculation involve parameter optimization in generating the final prediction model and are thus vulnerable to overfitting [14]. The best strategy to avoid overfitting is to evaluate performance in an independent validation sample, but such a sample is not always available. Alternatively, if the primary aim is to assess evidence for an association to test a hypothesis, then we can calculate an empirical *P*-value corresponding to the association of the optimized PRS, with the Type 1 error rate controlled [13].

In PRSice-2, to obtain the empirical *P*-value, the target trait values are permuted across the sample of individuals *k* times (default = 10,000) and the PRS analysis is repeated on each set of permuted phenotypes. Thus, on each permutation, the “best-fit PRS” is obtained as that most associated with the target trait across the range of *P*-value thresholds considered, and the empirical *P*-value is calculated as:

(2) \begin{equation*} \mathrm{empirical}\ P\ = \ \frac{{\mathop \sum \nolimits_{n\ = \ 1}^N I({P_n} < {P_o}) + 1}}{{N + 1}}, \end{equation*}

where *N* is the number of permutations performed; $I\left(.\right)$is an indicator function, which takes a value of 1 if the *P*-value of the best-fit PRS of permutation *n* is smaller than the observed *P*-value, *P_o_*, and 0 otherwise; and where pseudo-counts of 1 are added to the numerator and denominator to avoid empirical *P*-values of 0 and reflecting (conservatively) counting the observed trait configuration as 1 potential null permutation [22]. While the empirical *P*-values for association will be controlled for the Type 1 error rate, because the same process of parameter optimization is performed explicitly under the null hypothesis, the observed phenotypic variance explained, *R*^2^, remains unadjusted and is affected by overfitting. Therefore, it is imperative to perform out-of-sample prediction, or cross-validation, to evaluate the predictive accuracy of PRS when using PRSice-2, and ideally the former given the problems of generalizability observed with PRS [14].

## Analysis of PRS Strata

While PRSs on most complex traits presently have limited power to accurately predict risk at the individual level, which will remain the case for low-to-moderate–heritability traits irrespective of GWAS sample sizes, recent studies have demonstrated that individuals at the tails of PRS distribution can have substantially higher disease risk than those of the general population. Thus, these individuals may provide useful subjects for experimental follow-up, while in clinical settings it could be more efficacious to use different risk management strategies, in terms of screening or interventions, for example, for individuals with extreme PRS [1–3].

**Figure 1.**
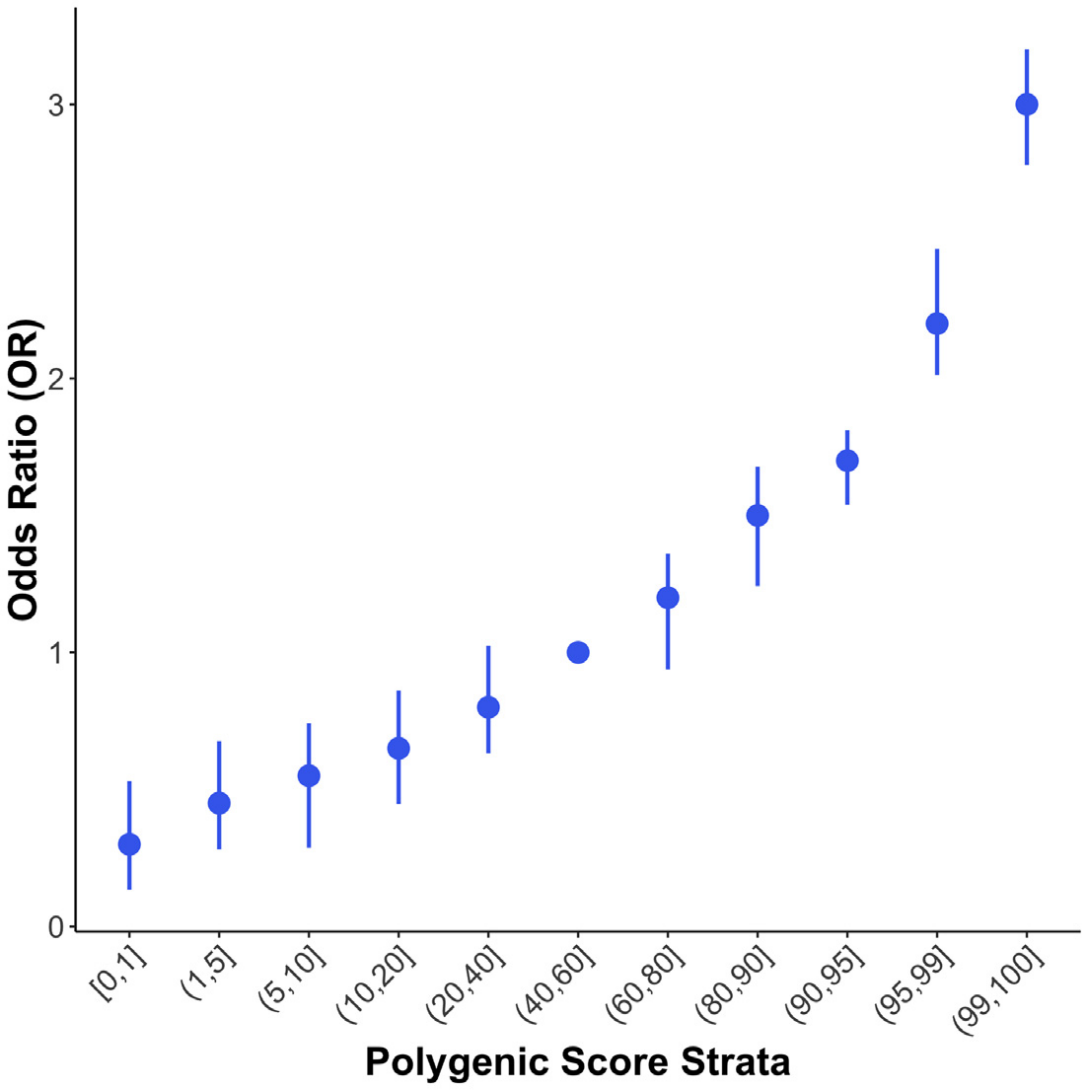
Strata plot generated by PRSice-2. The X-axis shows the range of different quantiles (e.g., (80,90] corresponds to those individuals with PRS between the 80th and 90th percentile of the population), and the Y-axis shows the odds ratio when comparing PRS from different quantiles with the reference quantile (here, (40,60]), with the bars corresponding to 95% confidence intervals of the odds ratio.

We have implemented a strata analysis feature in PRSice-2 to aid the calculation of relative phenotypic risk of individuals between strata. Briefly, the *N* individuals of the target sample are first aggregated into *M* different strata based on their PRS. An *N* x (M − 1) design matrix is then generated using dummy coding, such that an individual is coded 1 in the column that corresponds to their PRS stratum and whereby a user-defined stratum is the reference group (or the median stratum by default). A linear regression (for quantitative traits) or logistic regression (for binary traits) will then be performed to estimate the phenotypic difference or relative risk, respectively, of each stratum vs the reference. The set of corresponding β-coefficients (linear) or the odds ratio (logistic) can then be visualized with the strata plot (Fig. 1). This allow users to assess whether individuals in the extreme stratum have a substantially higher phenotypic risk when compared to the reference stratum.

## Benchmarking

Here we perform a simulation study to compare the performance of PRSice-2 to alternative polygenic score software lassosum [15] and LDpred [16], in terms of run time, memory usage, and predictive power.

Quantitative traits with heritability (*h*^2^) of 0.2 and 0.6 were simulated with the UK Biobank genotype data (post–quality control) as input. Briefly, each quantitative trait was simulated on the basis of the following linear model:

(3) \begin{equation*} Y\ = {\rm{\ }}X\beta + \varepsilon, \end{equation*}

where *X* is the unstandardized genotype matrix corresponding to 385,794 individuals (rows) and 560,173 SNP genotypes (columns). The β vector corresponds to the effect size associated with each SNP, with 100, 1,000, 10,000, 100,000, and 560,173 (all SNPs) randomly selected to be causal SNPs with effect size $\beta \sim\,N( {0,1} )$, β = 0 otherwise, and $\varepsilon $ represents the random error, which follows $\varepsilon \sim N\left(0,\sqrt{var(X\beta)(1-h^2)/h^2}\right)$. To control for batch effects and population structure in the genotype data, a regression of batch and 40 Principal Components (PCs) against the simulated trait were performed as follows:

(4) \begin{equation*} Y\ = \mathrm{Batch} + 40 \ \mathrm{PCs} + \varepsilon. \end{equation*}

The standardized residuals were then used as the final simulated trait. Samples of size 50,000 and 200,000 individuals were randomly selected as the base sample and used to generate the GWAS summary statistics. Then 100, 1,000, 10,000, and 100,000 samples independent from the base were randomly selected as the target sample. PRS analyses were then performed on these base and target data using the latest version of lassosum (v0.4.4), LDpred (v1.0.6), and PRSice 2 (v2.2.1), on servers equipped with 286 Intel 8168 24 core processors at 2.7 GHz and 192 GB of RAM. Default parameters of each program were used. The run time and memory usage of each program were measured using the Linux "time" command and the predictive power of the methods was assessed according to phenotypic variance explained (*R*^2^). The entire process was repeated 10 times to obtain an estimated distribution of run time, memory usage, and predictive power.

**Figure 2.**
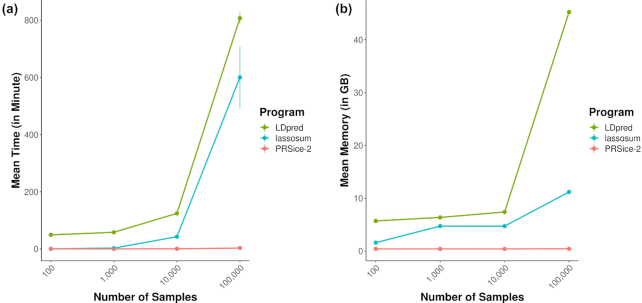
Performance of the 3 PRS software programs on simulated data. (a) Mean run time (in minutes) required to complete the entire analysis, across 10 repeats, when applied to different sizes of target sample. (b) Mean memory (in GB) required for the different software programs to process the different sizes of target sample.

Figure 2 shows the run time and memory usage of PRSice-2, lassosum, and LDpred. Based on these simulation results, PRSice-2 is the most efficient software in all settings (Fig. 2a), significantly faster than lassosum (*P* = 1e−58, 1-sided *t*-test) and LDpred (*P* = 2e−90, 1-sided *t*-test). Specifically, PRSice-2 can complete the full PRS analysis on 100,000 samples within 4 minutes (Supplementary Table 1), which is 179 times faster than the 10 hours required by lassosum and 241 times faster than the 13 hours 27 minutes required by LDpred. Likewise, PRSice-2 requires significantly less memory (Fig. 2b) than lassosum (*P* = 1e−202, 1-sided *t*-test) and LDpred (*P* = 9e−112, 1-sided *t*-test), requiring <500 MB of memory for 100,000 samples, as opposed to 11.2 GB required by lassosum and 45.2 GB required by LDpred (Supplementary Table 2). Likewise, PRSice-2 outperforms PRSice-v1.25, requiring 400 times less time and 8 times less memory for a target sample size of 10,000 (similar memory for small target samples; see Supplementary Fig. 1 and Supplementary Tables 1 and 2 for details). As data size grows, or when more sophisticated PRS analyses are performed at scale [5,23], these gains in computational efficiency could become even more important.

**Figure 3.**
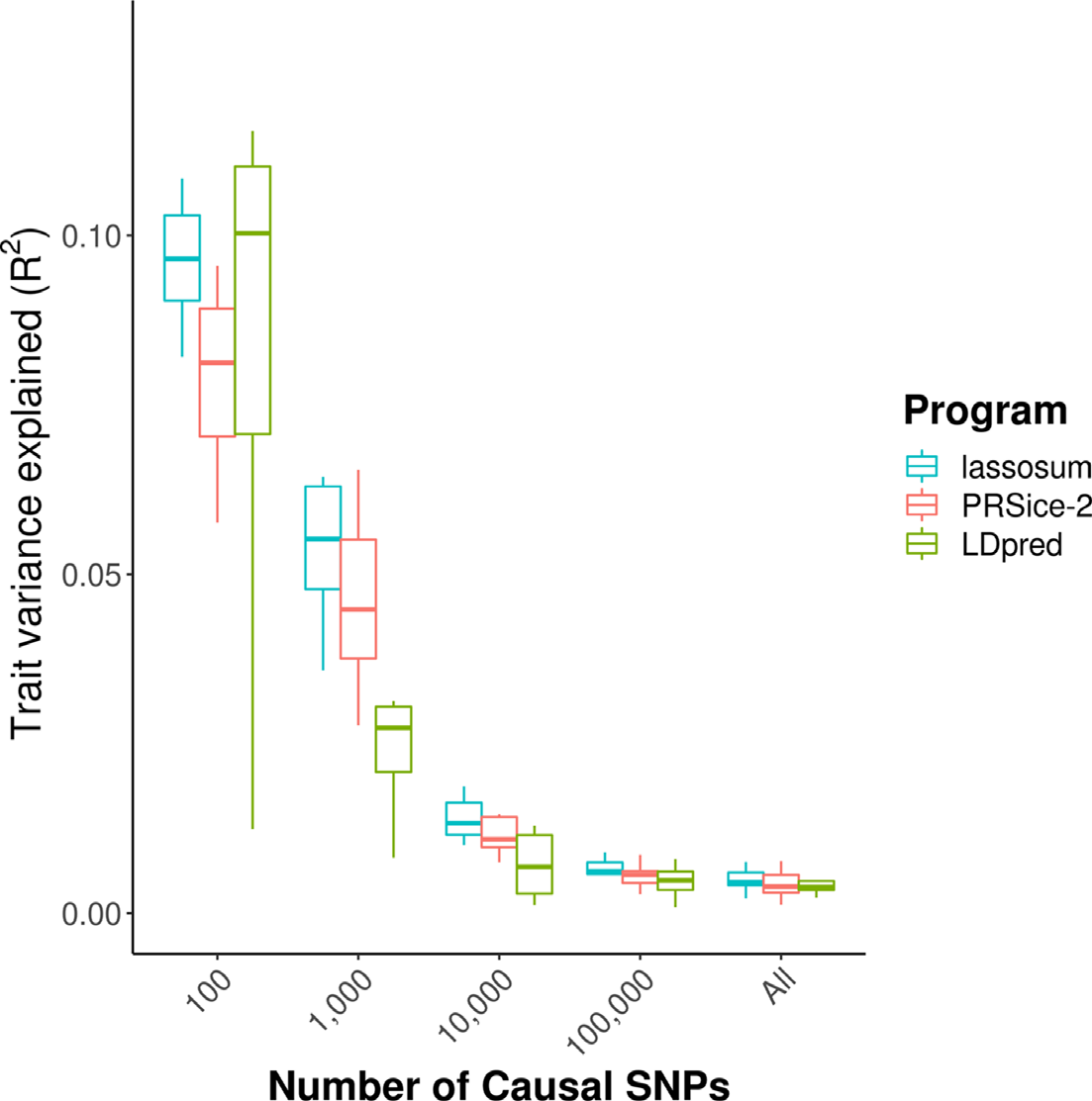
Predictive accuracy of the 3 PRS software programs for a simulated trait with heritability *h*^2^ = 0.2, target sample size of 10,000, and base sample size of 50,000. The 3 programs were run using their default parameter settings. The Y-axis represents the trait variance explained (*R*^2^) by the PRS generated from each software program, while the X-axis corresponds to the number of causal SNPs for the simulated trait. The horizontal line within boxes corresponds to the median values, while the lower and upper hinges correspond to the lower and upper quartiles, respectively, and the lines extend to the minimum and maximum values if those lie within 1.5 times the inter-quartile range (IQR); if not, then they extend to 1.5 times the IQR. Full results of the comparison study are shown in Supplementary Fig. 2.

Figure 3 shows the predictive power of PRSice-2 when compared to lassosum and LDpred for quantitative traits with heritability of 0.2, base sample size of 50,000, and target sample size of 10,000 (see Supplementary Fig. 2 for comparisons across all settings). Consistent with previous findings [15,24, 25], PRSice-2 has comparable predictive power to lassosum and LDpred, typically generating PRSs with predictive power higher than those of LDpred but not as high as lassosum. However, these results are inherently dependent on our modelling assumptions. For example, in our simulation, effect sizes and residual effects are assumed to have a Gaussian distribution and all “causal” SNPs are included in the dataset. Thus, we provide these results only as an approximate guide to performance in settings that match our assumptions. We provide our simulation code [26] for others to inspect and repeat our analyses.

While PRSs generated by PRSice-2 do not seem to fully optimize predictive accuracy, the simple approach and typically fewer SNPs exploited allow for easier interpretation of the results compared with methods that use all SNPs [27]. Moreover, the efficiency and predictive power of PRSice-2 make it an ideal tool to perform PRS analyses at scale.

## Discussion

We have introduced PRSice-2, a software program for the automation of PRS analyses applied to large-scale genotype-phenotype data. Our results demonstrate that PRSice-2 is the most efficient among leading PRS software, outperforming lassosum [15] and LDpred [16]. As data sizes increase and more sophisticated PRS analyses, such as multi-trait or gene set–based PRS analyses, become common, the efficiency advantages of PRSice-2 will become increasingly important.

Overfitting is a concern for all approaches to PRS analyses [14]. To control for the Type 1 error rate caused by overfitting when exploiting PRS for hypothesis testing, PRSice-2 implements the calculation of empirical *P*-values.

PRSice-2 implements a standard approach for performing PRS analyses. For PRS analyses performed in family data or across diverse populations, for instance, results should be interpreted carefully [14] and extensions of the standard PRS approach or alternatives may be required [14,28–30] to generate more informative results.

## Availability of supporting source code and requirements

Project Name: PRSice-2

Project home page: http://prsice.info

Operating systems (pre-compiled versions): Linux (64-bit), OS X (64-bit Intel), Windows (64-bit)

Programming language: C++, R (version 3.2.3+)

Other requirements (when recompiling): GCC version 4.8+, zlib

License: GNU General Public License version 3.0 (GPLv3)

Any restrictions to use by non-academics: None

RRID: SCR_01 7057

## Availability of supporting data and materials

Data further supporting this work and snapshots of the code are available in the *GigaScience* repository, GigaDB [31].

## Additional files

**Supplementary Figure 1**. Performance of PRSice-2 compared to PRSice-1.25. (a) Mean run time (in minutes) required to complete the entire analysis, across 10 repeats, when applied to different sizes of target sample. (b) Mean memory (in GB) required to process the different sizes of target sample.

**Supplementary Figure 2**. Predictive accuracy of the 3 PRS software programs across all simulated scenarios using the default parameters. The y-axis represents the trait variance explained (*R*^2^) by the PRS generated from each software program, while the x-axis corresponds to the number of causal SNPs for the simulated trait. The right side of the graph shows the number of base samples included in the simulation and heritability of the simulated trait while the top of the graph shows the number of target samples included in the simulation.

**Supplementary Table 1**. Mean run time (in minutes) for each program over the 10 iterations, across different heritability and number of causal SNPs. Standard error of run time is in brackets. Due to the large number of intermediate files generated by PRSice-1.25 and the excessive run time required, we did not test the run time of PRSice-1.25 with 100,000 target samples.

**Supplementary Table 2**. Mean memory usage (in GB) for each program over the 10 iterations, across different heritability and number of causal SNPs. Standard error of memory usage is in brackets. Due to the large number of intermediate files generated by PRSice-1.25 and the excessive run time required, we did not test the memory use of PRSice-1.25 with 100,000 target samples.

**Supplementary Note 1**: Quality Control of UK Biobank data.

giz082_GIGA-D-18-00468_Original_Submission

giz082_GIGA-D-18-00468_Revision_1

giz082_GIGA-D-18-00468_Revision_2

giz082_Response_to_Reviewer_Comments_Original_Submission

giz082_Response_to_Reviewer_Comments_Revision_1

giz082_Reviewer_1_Report_Original_Submission Farhad Hormozdiari -- 12/28/2018 Reviewed

giz082_Reviewer_1_Report_Revision_1 Farhad Hormozdiari -- 4/5/2019 Reviewed

giz082_Reviewer_2_Report_Original_Submission Daniel Belsky -- 1/15/2019 Reviewed

giz082_Reviewer_2_Report_Revision_1 Daniel Belsky -- 3/15/2019 Reviewed

giz082_Supplemental_File

## Abbreviations

BMI: body mass index; GWAS: genome-wide association study; PRS: polygenic risk score; RAM: random access memory; SNP: single-nucleotide polymorphism; VCF: Variant Call Format.

## Competing interests

The authors declare that they have no competing interests.

## Funding

Medical Research Council FundRef identification ID: http://dx.doi.org/10.13039/501100000265 MR/N015746/1 to P.F.O. S.W.C. is funded from the UK Medical Research Council (MR/N015746/1). This report represents independent research (part)-funded by the National Institute for Health Research (NIHR) Biomedical Research Centre at South London and Maudsley NHS Foundation Trust and King's College London. The views expressed are those of the authors and not necessarily those of the NHS, the NIHR, or the Department of Health.

## Authors’ contributions

Conceptualization, S.W.C. and P.F.O.; Methodology, S.W.C. and P.F.O.; Investigation, S.W.C.; Software, S.W.C.; Supervision, P.F.O.; Funding Acquisition, P.F.O.; Writing—Original Draft, S.W.C.; Writing—Review and Editing, S.W.C. and P.F.O.
