# Supplementary material for: PRSice-2: Polygenic Risk Score software for biobank-scale data
Source: Gigascience. 2019 Jul 15;8(7):giz082. doi: 10.1093/gigascience/giz082 (PMC6629542; doi:10.1093/gigascience/giz082)
Supplement: giz082_GIGA-D-18-00468_Original_Submission [file giz082_giga-d-18-00468_original_submission.pdf]

GigaScience

PRSice-2: Next Generation Polygenic Risk Score Analysis Software

--Manuscript Draft--

**Manuscript Number:**	GIGA-D-18-00468
**Full Title:**	PRSice-2: Next Generation Polygenic Risk Score Analysis Software
**Article Type:**	Technical Note
**Funding Information:**	UK Medical Research Council (MR/N015746/1) — Dr Shing Wan Choi<br>Dr Paul F O'Reilly
**Abstract:**	Background<br><br>Polygenic Risk Score (PRS) analyses have become an integral part of biomedical research, exploited to gain insights into shared aetiology among traits, to control for genomic profile in experimental studies, and to strengthen causal inference, among a range of applications. Substantial efforts are now devoted to biobank projects to collect large genetic and phenotypic data, providing unprecedented opportunity for genetic discovery and applications. To process the large-scale data provided by such biobank resources, highly efficient and scalable methods and software are required.<br><br>Method<br><br>Here we introduce PRSice-2, an efficient and scalable software for automating and simplifying polygenic risk score analyses on large-scale data. PRSice-2 handles both genotyped and imputed data, provides empirical association P-values free from overfitting effects, supports different inheritance models and can evaluate multiple continuous and binary target traits simultaneously. We demonstrate that PRSice-2 is significantly faster than alternative polygenic score software, LDpred and lassosum, which will be increasingly important as data sizes grow and as the applications of PRS become more sophisticated, e.g. when incorporated into high-dimensional or gene-set based analyses.<br><br>Conclusion<br><br>PRSice-2 is written in C++, with an R script for plotting, and is freely available for download from http://PRSice.info
**Corresponding Author:**	Shing Wan Choi<br>King's College London<br>London, UNITED KINGDOM
**Corresponding Author Secondary Information:**	
**Corresponding Author's Institution:**	King's College London
**Corresponding Author's Secondary Institution:**	
**First Author:**	Shing Wan Choi
**First Author Secondary Information:**	
**Order of Authors:**	Shing Wan Choi
	Paul F O'Reilly
**Order of Authors Secondary Information:**	
**Additional Information:**	
**Question**	**Response**
Are you submitting this manuscript to a special series or article collection?	No

Powered by Editorial Manager® and ProduXion Manager® from Aries Systems Corporation

**Experimental design and statistics**<br><br>Full details of the experimental design and statistical methods used should be given in the Methods section, as detailed in our Minimum Standards Reporting Checklist. Information essential to interpreting the data presented should be made available in the figure legends.<br><br>Have you included all the information requested in your manuscript?	Yes
**Resources**<br><br>A description of all resources used, including antibodies, cell lines, animals and software tools, with enough information to allow them to be uniquely identified, should be included in the Methods section. Authors are strongly encouraged to cite Research Resource Identifiers (RRIDs) for antibodies, model organisms and tools, where possible.<br><br>Have you included the information requested as detailed in our Minimum Standards Reporting Checklist?	Yes
**Availability of data and materials**<br><br>All datasets and code on which the conclusions of the paper rely must be either included in your submission or deposited in publicly available repositories (where available and ethically appropriate), referencing such data using a unique identifier in the references and in the "Availability of Data and Materials" section of your manuscript.<br><br>Have you have met the above requirement as detailed in our Minimum Standards Reporting Checklist?	Yes

Powered by Editorial Manager® and ProduXion Manager® from Aries Systems Corporation

 Click here to download Manuscript 20181123-PRSice2.docx

PRSice-2: Next Generation Polygenic Risk Score Analysis Software

Shing Wan Choi* and Paul O'Reilly

MRC Social, Genetic and Developmental Psychiatry Centre, Institute of Psychiatry, Psychology and Neuroscience, King's College London, London, United Kingdom

**To whom correspondence should be addressed.*

Abstract

Background: Polygenic Risk Score (PRS) analyses have become an integral part of biomedical research, exploited to gain insights into shared aetiology among traits, to control for genomic profile in experimental studies, and to strengthen causal inference, among a range of applications. Substantial efforts are now devoted to biobank projects to collect large genetic and phenotypic data, providing unprecedented opportunity for genetic discovery and applications. To process the large-scale data provided by such biobank resources, highly efficient and scalable methods and software are required.

Method: Here we introduce PRSice-2, an efficient and scalable software for automating and simplifying polygenic risk score analyses on large-scale data. PRSice-2 handles both genotyped and imputed data, provides empirical association *P*-values free from overfitting effects, supports different inheritance models and can evaluate multiple continuous and binary target traits simultaneously. We demonstrate that PRSice-2 is significantly faster than alternative polygenic

score software, LDpred and lassosum, which will be increasingly important as data sizes grow and as the applications of PRS become more sophisticated, eg. when incorporated into high-dimensional or gene-set based analyses.

Conclusion: PRSice-2 is written in C++, with an R script for plotting, and is freely available for download from http://PRSice.info

Keywords: Polygenic Risk Score, GWAS, Imputation

Polygenic Risk Score (PRS) analyses are beginning to play a critical role in biomedical research, proving to have both scientific and clinical utility [1–9]. The increasing availability of genetic data from regional and national biobank projects [10–12] have allowed more powerful PRS to be calculated. However, the calculation of PRS, which involves parameter optimization [13–16], can be a computationally intensive process, especially for large datasets and when multiple analyses are conducted. To fully utilize the power of large datasets and to facilitate future method and application developments, at scale, we have performed a major overhaul of our original PRSice software [13], to produce PRSice-2. All code has been re-written in C++ and code from PLINK-1.9 [17] that minimised computation has been incorporated. As a result of the consistent language and switch to objected-oriented code, different analytical components of the code can communicate directly, without, for example, the generation of intermediate files, such as those containing PRS corresponding to each *P*-value threshold, or post-processed genotype files. This has generated a substantial speed-up and reduction in disk space requirement in PRSice-2. In addition, a separate plotting script was implemented in R. Separate tasks are organized into functions and are, thus, more amenable to tailored extensions by users. Finally, a range of user-options were incorporated into PRSice-2 to increase flexibility and improve usability.

Features of PRSice-2

PRSice-2 retains the majority of the features of its predecessor PRSice [13], including automatic strand flipping, single nucleotide polymorphism (SNP) thinning according to linkage disequilibrium (LD) and *P*-value, known as clumping [18], and calculation and evaluation of PRS under few (‘fastscore’) or many (‘high-resolution scoring’) *P*-value thresholds.

When compared to PRSice, PRSice-2 streamlines the entire PRS analysis pipeline without generating intermediate files, and performs all the main computations in C++, leading to a drastic speed-up in runtime and reduction of storage space. Extraction and exclusion of samples and SNPs are also implemented, allowing PRS analysis to be performed directly on a subset of the input data without performing pre-filtering.

Briefly, the main new features of PRSice-2 are:

1. Handles large-scale PRS analyses of both genotyped and imputed data.
2. Computes empirical association *P*-values to account for over-fitting.
3. Can perform PRS analyses on extensive number of target phenotypes simultaneously.
4. Provides several options for imputing missing genotypes.
5. Allows calculation of PRS based on different inheritance models, including additive, dominant, recessive and heterozygous models.
6. Automatically generates dummy variables for categorical covariates.
7. Can perform regression to estimate relative effect/risk corresponding to samples in user-defined stratum of the population. Can output quantile and strata plots.
8. Amenable to user extensions, such as relating to input data format, regression modelling and output.

Handling of Imputed data

Genotypes are typically represented as the discrete counts of the minor or effect allele (0, 1 or 2), for single nucleotide polymorphisms (SNPs), in each individual. Genotypes not included in the genotyping chip can, potentially, be imputed and are usually either recorded as a set of three probabilities corresponding to the probability of each of the possible genotypes [19], or based on these, as the expected genotype (a real number between 0 and 2 known as the "dosage") [19] or as the "best guess" (most probable) genotype. While any of these data formats can be exploited in PRS analyses, the most common approach is to use the "best-guess" genotype for each individual. However, this approach ignores the uncertainty in the imputed genotype.

Currently, most PRS software only support input of the genotyped format. Therefore, users need to generate a large intermediate file containing the best-guess genotypes and discard any information related to imputation uncertainty. To reduce the storage space requirement, and to incorporate imputation uncertainty into PRS analyses, PRSice-2 implements support for the BGEN imputation format. PRSice-2 can directly process the BGEN imputed format and either convert to best-guess genotypes or dosages when calculating the PRS, without generating a large intermediate file. While PRS based on best-guess genotypes are calculated as for genotyped input, dosage based PRS are calculated as

$$PRS = \left(\sum_{i}^{m} \beta_i \left(\sum_{j=0}^{2} \omega_{ij} \times j\right)\right) \quad (1)$$

where ω_{ij} is the probability of observing genotype j , where $j \in \{0,1,2\}$, for the ith SNP, m is the number of SNPs and β_i is the effect size of the ith SNP estimated from the relevant base GWAS data.

The ability to perform PRS analyses directly on imputed data can be particularly useful when the base GWAS and target samples are genotyped on a different platforms, as then there can be a small fraction of overlapping SNPs. For example, of the 725,459 post-QC SNPs (see Supplementary Material) in the UK Biobank genotype data [10], only 31% (222,956) of those were found in the GIANT Height and Body Mass Index (BMI) GWAS [20,21]. The use of imputed SNPs increases the number of overlapping SNPs to 2,121,036 SNPs. To assess the gain of power when using imputed vs un-imputed data, we performed PRS analyses on Height and BMI using UK Biobank genotyped and imputed data, with GWAS summary statistics provided by the GIANT consortium [20,21]. Age, UK Biobank genotyping batch, UK Biobank assessment centre and 40 principle components were first regressed out from the phenotype and the standardized residuals were used instead. In the linear regression, performed by PRSice-2 in the UK Biobank data as target sample using the default parameters, with height as outcome and PRS for height as predictor, we observed an increase in phenotypic variance explained (R^2) by the PRS from 0.141 (genotyped) to 0.152 (dosage), and likewise for BMI of 0.0456 to 0.0535.

However, a challenge with imputed data is that there are numerous imputed formats in the field. While it is difficult to support all imputed formats, PRSice-2 adopts a modular approach, which allows simple incorporation of supports for additional data formats (eg. vcf) in the future.

Calculation of Empirical *P*-value

All approaches to PRS calculation involve parameter optimisation in generating the final prediction model, and are thus vulnerable to overfitting [14]. The best strategy to avoid overfitting is to evaluate performance in an independent validation sample, but such a sample is not always available. Alternatively, if the primary aim is to assess evidence for an association to test a hypothesis, then we can calculate an empirical *P*-value corresponding to the association of the optimized PRS, with the Type 1 error rate controlled [13].

In PRSice-2, to obtain the empirical *P*-value, the target trait values are permuted across the sample of individuals k times (default = 10,000) and the PRS analysis repeated on each set of permuted phenotypes. Thus, on each permutation, the "best-fit PRS" is obtained as that most associated with the target trait across the range of *P*-value thresholds considered, and the empirical *P*-value is calculated as:

$$empirical\ P = \frac{\sum_{n=1}^{N} I(P_n < P_o) + 1}{N + 1} \qquad (2)$$

where N is the number of permutations performed, $I(.)$ is the indicator function, which takes a value of 0 if the "best-fit PRS" of permutation n is smaller than the observed *P*-value, P_o, and where pseudo-counts of 1 are added to the numerator and denominator to avoid empirical *P*-values of 0 and reflecting (conservatively) counting the observed trait configuration as one potential null permutation [22]. While the empirical *P*-values for association will have controlled for the Type 1 error rate, since the same process of parameter optimisation is performed explicitly under the null hypothesis, the observed phenotypic variance explained R^2 remain unadjusted and affected by overfitting. Therefore, it is imperative to perform out-of-sample prediction, or cross-validation, to

evaluate the predictive power of PRS when using PRSice-2, and ideally the former given the problems of generalisability observed with PRS [14].

Analysis of PRS strata

While PRS on most complex traits presently have limited power to predict individual risk across the population, which will remain limited for low-moderate heritability traits irrespective of GWAS sample sizes, recent studies have demonstrated that individuals at the tails of PRS distribution have substantially higher disease risk compared to those of the general population. Thus, it could be more efficacious to employ a different risk management strategy, in terms of screening or interventions, for example, to individuals with extreme PRS [1–3].

We implemented the strata analysis feature in PRSice-2 to assist the calculation of relative phenotypic risk of individuals within different strata. Briefly, assuming there are N individuals, they will first be aggregated into M different strata based on their PRS. A M row by $N-1$ column design matrix were then generated using dummy coding, using a user defined stratum as the reference group (or the median stratum by default). A linear regression (for quantitative traits) or logistic regression (for binary traits) will then be performed to obtain the relative phenotypic risk of each stratum against the reference, represented by the beta-coefficient (or the odds ratio for binary outcome, which can then be visualized using the strata plot (Figure 1). This allow users to test whether individuals at the extreme stratum have a substantially higher phenotypic risk when compared to the reference stratum.

Figure 1

Figure 1 Strata plot generated by PRSice-2. The X-axis shows the range of different quantiles (eg. (80,90] corresponds to those individuals with PRS between the 80%-ile – 90%-ile of the population), and the Y-axis shows the coefficient of regression when comparing PRS from different quantiles with the reference quantile (here, (40,60]).

Benchmarking

PRSice-2 utilizes the standard approach to PRS calculation involving clumping SNPs and then performing the *P*-value thresholding strategy, known as the "C+T" method [14]. Studies [15,23] have shown that this approach has comparable predictive power to more complex methods such as lassosum [15] and LDpred [16]. As data size grows, or when more sophisticated PRS analyses are performed at scale [5,24], then computational efficiency becomes more important.

Here, we compared the runtime and memory usage of PRSice-2 versus lassosum [15] and LDpred [16]. We simulated a phenotype for each individual in the UK biobank based on genetic effect sizes drawn from a standard normal distribution plus error. 100, 1k, 10k and 100k samples were then randomly selected from the UK biobank and used as the target data. PRS analyses were then performed using lassosum (v0.4.1), LDpred (v0.9.1) and PRSice 2 (v2.1.4), on servers equipped with two 10 core Intel Haswell E5-2660 v3 @ 2.60GHz and 128GB of RAM. Default parameters of each program were used. Runtime and memory usage of each program were measured using the Linux *time* command. The entire process was repeated 5 times to obtain an estimated distribution of runtime and memory usage.

Our simulation results demonstrated that PRSice-2 is the most efficient software in all settings (Figure 2a) and that the memory usage scales well with the number of samples (Figure 2b). Specifically, PRSice-2 can complete the full PRS analysis on 100k samples within an average of 8 minutes (Supplementary Table 1), significantly faster than lassosum (P = 2.5e-6, two tailed t-

test), which takes an average 6 hours 13 minutes, and LDpred (*P* = 7.2e-5, two tailed t-test), which takes approximately 19 hours. Similarly, with 100k target samples, PRSice-2 requires less than 600MB of memory (Supplementary Table 2), which is significantly less than the 7.35 Gb required by lassosum (*P* = 9.6e-12, two tailed t-test) and the 51.2 Gb required by LDpred (*P* = 1.7e-34, two tailed t-test). With its quick runtime and low memory usage, PRSice-2 can perform PRS analyses at scale on a desktop computer.

Figure 2a	Figure 2b

Figure 2 Performance of the three PRS software. a) Average run time (in minutes) required to complete the whole analysis when different number of target samples were used. B) Average memory (in GB) required for the software to process different number of target samples.

Discussion

We have introduced PRSice-2, a software for the automation of polygenic risk score (PRS) analyses in large-scale genetic-phenotype data. Our results demonstrates that PRSice-2 is the most efficient among the leading PRS software, outperforming lassosum [15] and LDpred [16]. As data sizes increase and more complicated PRS analyses, such as multi-trait or gene-set based PRS analyses, become common, the efficiency advantages of PRSice-2 will become increasingly important.

Over-fitting is a concern for all approaches to PRS analysis [14]. To control for the Type 1 error rate caused by over-fitting when exploiting PRS for hypothesis testing, PRSice-2 implements the calculation of empirical *P*-values.

Availability and requirements

**Project Name**	PRSice-2
**Project home page**	http://prsice.info
**Operating systems (pre-compiled versions)**	Linux (64-bit)<br>OS X (64-bit Intel)<br>Windows (64-bit)
**Programming language**	C++, R (version 3.2.3+)
**Other requirements (when recompiling)**	GCC version 4.8+, zlib
**License**	GNU General Public License version 3.0 (GPLv3)
**Any restrictions to use by non-academics**	None

Declarations

Acknowledgements

We thank the participants in the UK Biobank and the scientists involved in the construction of this resource. This research has been conducted using the UK Biobank Resource under application 18177 (Dr O'Reilly). We thank Hei Man Wu for providing critical feedback regarding this manuscript and for test running the software. We thank Jonathan Coleman and Kylie Glanville for the management of the UK Biobank resource at King's College London, and we thank Jack

Euesden for his work on PRSice, which forms the basis of the current software. We thank Christopher Hübel, Eva Krapohl, Kirstin Purves, Jessye Maxwell, Saskia Hagenaars and Yunfeng Ruan for their help in test running the software. PFO receives funding from the UK Medical Research Council (MR/N015746/1). SWC is funded from the UK Medical Research Council (MR/N015746/1). This report represents independent research (part)-funded by the National Institute for Health Research (NIHR) Biomedical Research Centre at South London and Maudsley NHS Foundation Trust and King's College London. The views expressed are those of the authors and not necessarily those of the NHS, the NIHR, or the Department of Health.

Competing Interests

The authors declare that they have no competing interests

Authors' contributions

SWC and PFO designed the software. SWC implemented the software and drafted the manuscript. PFO provided critical feedback regarding the manuscript and the software development. All authors read and approved the final manuscript.

References

1. Mavaddat N, Pharoah PDP, Michailidou K, Tyrer J, Brook MN, Bolla MK, et al. Prediction of Breast Cancer Risk Based on Profiling With Common Genetic Variants. JNCI J Natl Cancer Inst [Internet]. 2015 [cited 2017 Jun 13];107. Available from: https://academic.oup.com/jnci/article/107/5/djv036/891009/Prediction-of-Breast-Cancer-Risk-Based-on

2. Kuchenbaecker KB, McGuffog L, Barrowdale D, Lee A, Soucy P, Healey S, et al. Evaluation of Polygenic Risk Scores for Breast and Ovarian Cancer Risk Prediction in BRCA1 and BRCA2 Mutation Carriers. JNCI J Natl Cancer Inst [Internet]. 2017 [cited 2018 Sep 26];109. Available from: https://www.ncbi.nlm.nih.gov/pmc/articles/PMC5408990/

3. Natarajan P, Young R, Stitziel NO, Padmanabhan S, Baber U, Mehran R, et al. Polygenic Risk Score Identifies Subgroup with Higher Burden of Atherosclerosis and Greater Relative Benefit

from Statin Therapy in the Primary Prevention Setting. Circulation. 2017;CIRCULATIONAHA.116.024436.

4. Udler MS, Kim J, Grotthuss M von, Bonas-Guarch S, Mercader JM, Cole JB, et al. Clustering of Type 2 Diabetes Genetic Loci by Multi-Trait Associations Identifies Disease Mechanisms and Subtypes. bioRxiv. 2018;319509.

5. Krapohl E, Euesden J, Zabaneh D, Pingault J-B, Rimfeld K, von Stumm S, et al. Phenome-wide analysis of genome-wide polygenic scores. Mol Psychiatry. 2016;21:1188–93.

6. Krapohl E, Patel H, Newhouse S, Curtis CJ, Stumm S von, Dale PS, et al. Multi-polygenic score approach to trait prediction. Mol Psychiatry. 2018;23:1368–74.

7. Selzam S, Krapohl E, von Stumm S, O’Reilly PF, Rimfeld K, Kovas Y, et al. Predicting educational achievement from DNA. Mol Psychiatry. 2017;22:267–72.

8. Selzam S, Dale PS, Wagner RK, DeFries JC, Cederlöf M, O’Reilly PF, et al. Genome-Wide Polygenic Scores Predict Reading Performance Throughout the School Years. Sci Stud Read. 2017;21:334–49.

9. Du Rietz E, Coleman J, Glanville K, Choi SW, O’Reilly PF, Kuntsi J. Association of Polygenic Risk for Attention-Deficit/Hyperactivity Disorder With Co-occurring Traits and Disorders. Biol Psychiatry Cogn Neurosci Neuroimaging. 2018;3:635–43.

10. Sudlow C, Gallacher J, Allen N, Beral V, Burton P, Danesh J, et al. UK Biobank: An Open Access Resource for Identifying the Causes of a Wide Range of Complex Diseases of Middle and Old Age. PLOS Med. 2015;12:e1001779.

11. Danciu I, Cowan JD, Basford M, Wang X, Saip A, Osgood S, et al. Secondary use of clinical data: The Vanderbilt approach. J Biomed Inform. 2014;52:28–35.

12. Kaiser J. NIH’s 1-million-volunteer precision medicine study announces first pilot projects. Science [Internet]. 2016 [cited 2018 Nov 15]; Available from: https://www.sciencemag.org/news/2016/02/nih-s-1-million-volunteer-precision-medicine-study-announces-first-pilot-projects

13. Euesden J, Lewis CM, O’Reilly PF. PRSice: Polygenic Risk Score software. Bioinformatics. 2015;31:1466–8.

14. Choi SW, Mak TSH, O’Reilly P. A guide to performing Polygenic Risk Score analyses. bioRxiv. 2018;416545.

15. Mak TSH, Porsch RM, Choi SW, Zhou X, Sham PC. Polygenic scores via penalized regression on summary statistics. Genet Epidemiol. 2017;41:469–80.

16. Vilhjálmsson BJ, Yang J, Finucane HK, Gusev A, Lindström S, Ripke S, et al. Modeling Linkage Disequilibrium Increases Accuracy of Polygenic Risk Scores. Am J Hum Genet. 2015;97:576–92.

17. Chang CC, Chow CC, Tellier LC, Vattikuti S, Purcell SM, Lee JJ. Second-generation PLINK: rising to the challenge of larger and richer datasets. GigaScience. 2015;4:7.

18. Wray NR, Lee SH, Mehta D, Vinkhuyzen AAE, Dudbridge F, Middeldorp CM. Research review: Polygenic methods and their application to psychiatric traits. J Child Psychol Psychiatry. 2014;55:1068–87.

19. Li Y, Willer C, Sanna S, Abecasis G. Genotype Imputation. Annu Rev Genomics Hum Genet. 2009;10:387–406.

20. Wood AR, Esko T, Yang J, Vedantam S, Pers TH, Gustafsson S, et al. Defining the role of common variation in the genomic and biological architecture of adult human height. Nat Genet. 2014;46:1173–86.

21. Locke AE, Kahali B, Berndt SI, Justice AE, Pers TH, Day FR, et al. Genetic studies of body mass index yield new insights for obesity biology. Nature. 2015;518:197–206.

22. North BV, Curtis D, Sham PC. A Note on the Calculation of Empirical P Values from Monte Carlo Procedures. Am J Hum Genet. 2002;71:439–41.

23. Allegrini A, Selzam S, Rimfeld K, Stumm S von, Pingault J-B, Plomin R. Genomic prediction of cognitive traits in childhood and adolescence. bioRxiv. 2018;418210.

24. Hagenaars SP, Harris SE, Davies G, Hill WD, Liewald DCM, Ritchie SJ, et al. Shared genetic aetiology between cognitive functions and physical and mental health in UK Biobank (N=112 151) and 24 GWAS consortia. Mol Psychiatry. 2016;21:1624–32.

Figure 1 Click here to download Figure Strata Plot.png

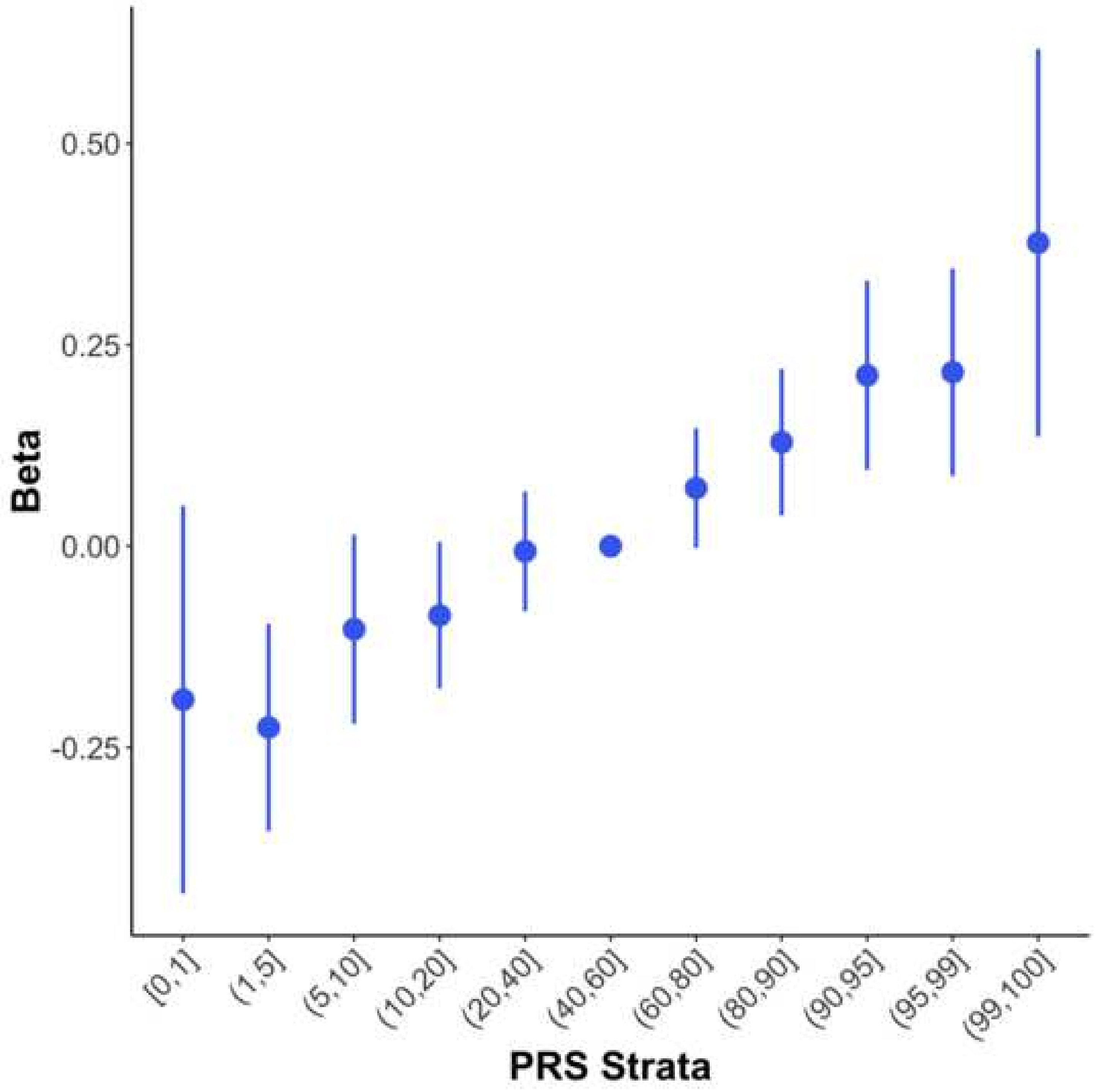

Figure 2a

Click here to download Figure Time.target.png

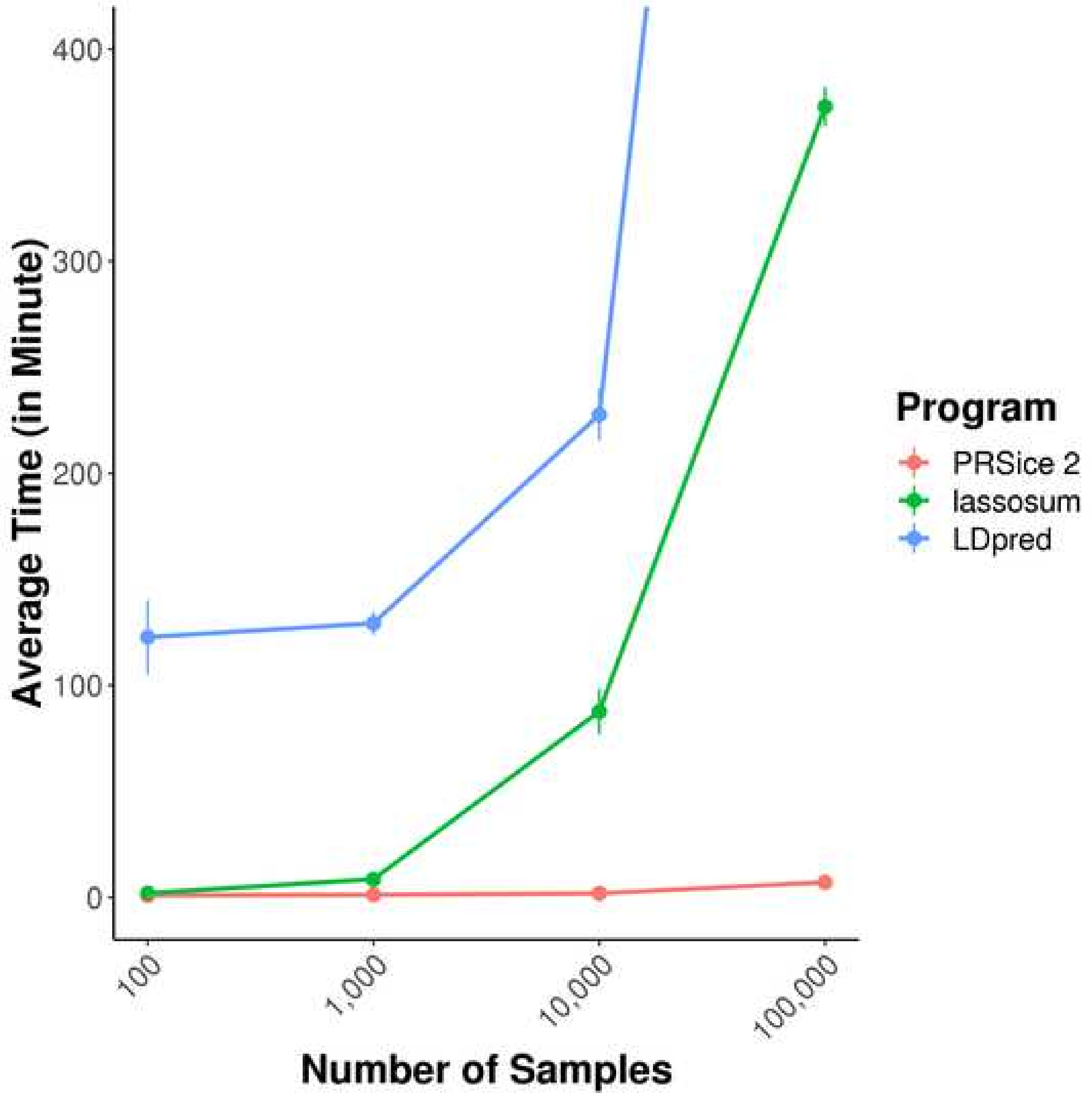

Figure 2b

Click here to download Figure Memory.target.png

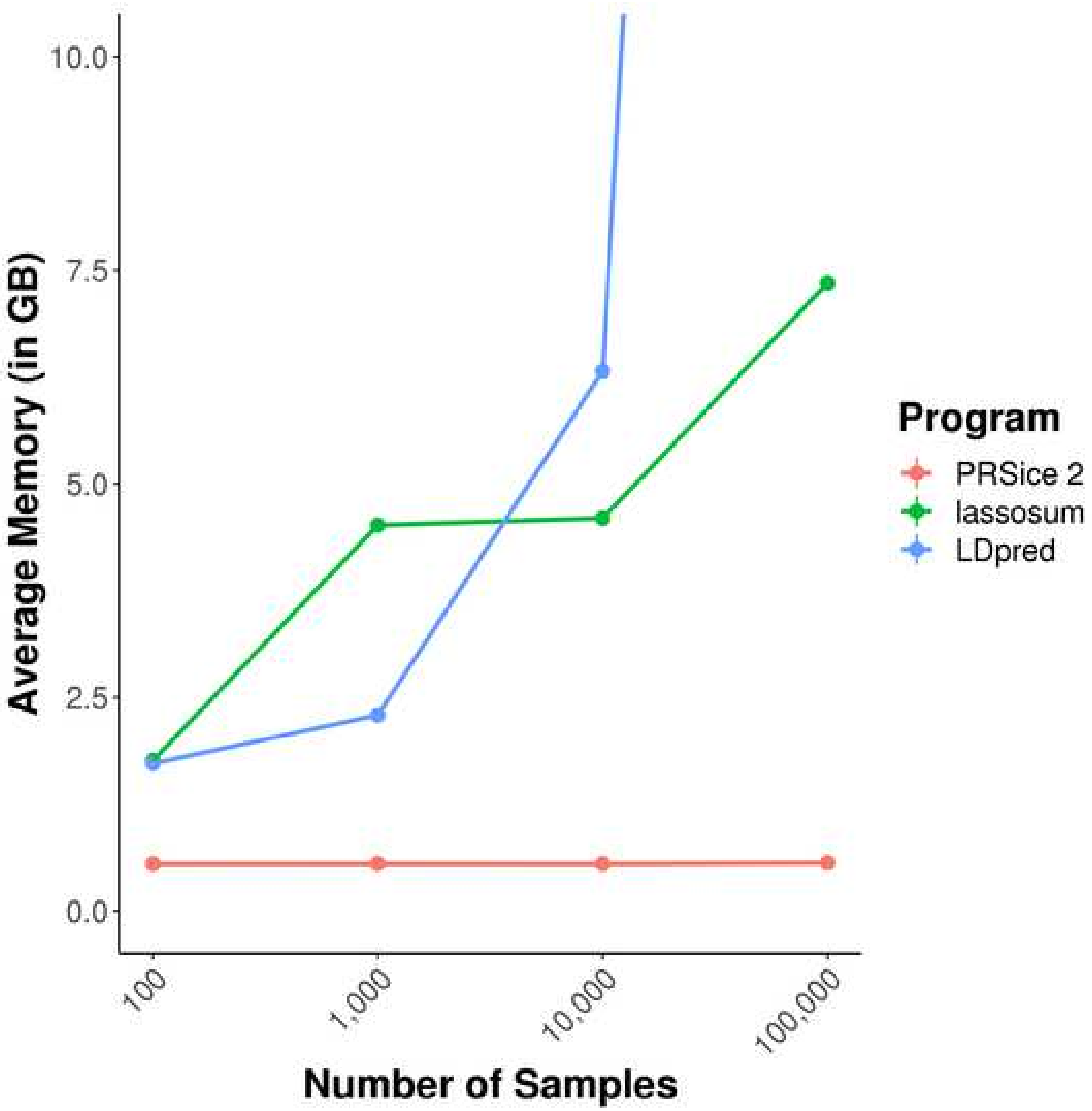

Click here to access/download
Supplementary Material
PRSice2 Supplementary.docx

Personal Cover
Click here to download Personal Cover
CoverLetter_PRSice2.docx

MRC Social, Genetic &
Developmental Psychiatry Centre
Director Francesca Happé

16 De Crespigny Park
Denmark Hill
London SE5 8AF

Dr Shing Wan Choi
Email: shing_wan.choi@kcl.ac.uk
Telephone: +44 (0)7729246486

A guide to performing polygenic risk score analyses

(For submission as a Technical Note)

27th November 2018

Dear Editor

Polygenic Risk Score (PRS) analyses have become an integral part of biomedical research, with promising clinical and scientific utility. Substantial efforts are now devoted to biobank projects to collect large genetic and phenotypic data, providing unprecedented opportunity for genetic discovery and application. However, the increased data size poses a substantial computational challenge to existing PRS tools, calling for the development of more efficient and scalable software.

Here, we present PRSice-2, a complete overhaul of our popular PRS software PRSice (Euesden et al. 2015; 286 citations, 150 citations in 2018). We have re-written the PRSice code in C++, making all code class/function based and thus more amenable to (user) extensions, have incorporated parts of the high performance PLINK-1.9 (Chang et al. 2015) algorithm where optimal, have extended data format options (eg. to BGEN), and via dramatic speed-ups and reductions in disk space requirement have made PRSice-2 now suitable for biobank scale data. A range of user-options and new features were also implemented in PRSice-2, providing increased flexibility and improved usability.

We present a performance comparison, demonstrating that PRSice-2 has a superior runtime compared to other leading PRS software, lassosum (Mak et al. 2017) and LDpred (Vilhjálmsson et al. 2015), having 45x and 143x faster runtime for PRS analyses performed on 10k samples. For the same data, PRSice-2 only requires 563Mb of memory, 13x less than the 7.35Gb required by lassosum and 90x less than the 51.2Gb required by LDpred. With its quick runtime and low memory usage, PRSice-2 can perform PRS analyses at scale on a desktop computer.

PRSice-2 is an open-source software, under GPL-3.0 license, with clear documentation (https://goo.gl/MFNvZX) and active support (https://goo.gl/Bb4hDT), making PRSice-2 arguably the most user-friendly PRS software. Given the popularity of PRSice, and the efficiency and functionality improvements of PRSice-2, we believe that our release and description of PRSice-2 would be ideally suited to GigaScience as a 'Technical Note'. We look forward to hearing back from you on this

Shing Wan Choi (cc'ed Paul F. O'Reilly)
