# Supplementary material for: PRSice-2: Polygenic Risk Score software for biobank-scale data
Source: Gigascience. 2019 Jul 15;8(7):giz082. doi: 10.1093/gigascience/giz082 (PMC6629542; doi:10.1093/gigascience/giz082)
Supplement: giz082_GIGA-D-18-00468_Revision_1 [file giz082_giga-d-18-00468_revision_1.pdf]

GigaScience

PRSice-2: Polygenic Risk Score Software for Large-Scale Data

--Manuscript Draft--

**Manuscript Number:**	GIGA-D-18-00468R1
**Full Title:**	PRSice-2: Polygenic Risk Score Software for Large-Scale Data
**Article Type:**	Technical Note
**Funding Information:**	UK Medical Research Council (MR/N015746/1) — Dr Shing Wan Choi<br>Dr Paul F O'Reilly
**Abstract:**	Background<br><br>Polygenic Risk Score (PRS) analyses have become an integral part of biomedical research, exploited to gain insights into shared aetiology among traits, to control for genomic profile in experimental studies, and to strengthen causal inference, among a range of applications. Substantial efforts are now devoted to biobank projects to collect large genetic and phenotypic data, providing unprecedented opportunity for genetic discovery and applications. To process the large-scale data provided by such biobank resources, highly efficient and scalable methods and software are required.<br><br>Method<br><br>Here we introduce PRSice-2, an efficient and scalable software for automating and simplifying polygenic risk score analyses on large-scale data. PRSice-2 handles both genotyped and imputed data, provides empirical association P-values free from inflation due to overfitting, supports different inheritance models and can evaluate multiple continuous and binary target traits simultaneously. We demonstrate that PRSice-2 is dramatically faster and more memory-efficient than PRSice and alternative polygenic score software, LDpred and lassosum, while having comparable predictive power. This combination of efficiency and power will be increasingly important as data sizes grow and as the applications of PRS become more sophisticated; for example, when incorporated into high-dimensional or gene-set based analyses.<br><br>Conclusion<br><br>PRSice-2 is written in C++, with an R script for plotting, and is freely available for download from http://PRSice.info
**Corresponding Author:**	Paul O'Reilly<br><br>UNITED KINGDOM
**Corresponding Author Secondary Information:**	
**Corresponding Author's Institution:**	
**Corresponding Author's Secondary Institution:**	
**First Author:**	Shing Wan Choi
**First Author Secondary Information:**	
**Order of Authors:**	Shing Wan Choi<br>Paul F O'Reilly
**Order of Authors Secondary Information:**	
**Response to Reviewers:**	Reviewer #1: Choi et al. have proposed a new extension of their PRSice method. The new method, PRSice-2, main advantage is speed as most of the code is written in C++ and PRSice-2 avoid creating intermediate files.

Powered by Editorial Manager® and ProduXion Manager® from Aries Systems Corporation

Major Comments:

1. The authors claim that their method is faster and more memory efficient than LDpred and lassosum. However, the authors need to compare these methods in case of prediction accuracy as well.

>> Thank you for your suggestion, which we think has now made our Technical Note more comprehensive. We have now included a full simulation analysis investigating the predictive accuracy of PRSice-2 compared to LDpred and lassosum (see Figure 3 and Supplementary Figure 2).

2. I like to see experiments where the authors compare PRSice-2 with PRSice performance.

>> We have now performed a comparison between PRSice-2 and PRSice-v1.25, both in terms of speed and memory (predictive accuracy is the same given the same underlying approach). Results can be found in Supplementary Figure 1, Supplementary Table 1 and Supplementary Table 2

Minor Comments:

The authors need to comment regarding the case where we have multiple populations in a study. For example Luna et al. Genetic epidemiology 2017 work discuss how to solve this problem.

The authors need to mention some of their method limitations in the discussion section.

>> Thank you for your comment. We agree that differences in allele frequencies, linkage disequilibrium and factors such as genetic drift and natural selection between populations can reduce the generalisability of PRS analyses across populations and produce misleading results, as suggested by Martin et. al. (2017) and as described in our 'Guide to performing polygenic risk score analyses' (Choi, Mak, O'Reilly. 2018. bioRxiv). We have now described this issue in our discussion, citing Duncan et al, Luna et al, Martin et al and Choi et al, and we caution users to take extra care when performing cross-population and family-wise PRS analyses.

Reviewer #2: This article reports the release of a new version of the PRSice software for polygenic score calculation. The new version of the software boasts speed enhancements that make it appealing for applications in the growing number of ultra-large genetically-informed datasets including the UK Biobank, 23andMe and others. Also important are features allowing for polygenic score computation from imputed genotype datasets in which genotypes are represented as a probabilities rather than discrete allele counts.

The data on speed are compelling. This alone is a good argument for why PRSice v1 users should upgrade to v2. But I found the article thinner on two other key questions central to addressing whether those not already using PRSice v2 should take up PRSice v2:
(1) Does the polygenic scoring method implemented within PRSice2 (additive combination of SNPs with/without LD clumping) deliver comparably predictive scores to other software, e.g. the LDPred and lassosum softwares?

>> Thank you for your comment and we agree that this is an important question. To address this, we have now performed a comprehensive simulation analysis to demonstrate the predictive power of PRSice-2 Vs LDpred and lassosum (see Figure 3 and Supplementary Figure 2).

(2) What is the value added of being able to accommodate imputed genotype probabilities rather than relying exclusively on discrete allele count data?

>> We thank the reviewer for this comment. We have now also performed an analysis to compare the predictive power of PRS constructed from genotyped data, or from imputed data either in terms of best-guess genotypes or dosage values. Briefly, the R2 for the Height PRS increased from 0.145 when using genotyped data to 0.152 when

Powered by Editorial Manager® and ProduXion Manager® from Aries Systems Corporation

	using best-guess imputed genotypes, and to 0.153 when using dosage data; likewise the R2 for BMI increased from 0.0475 when using genotype data to 0.0529 when using best-guess genotypes, and to 0.0535 when using dosage data.<br><br>I would suggest the following revisions:<br><br>Re PRSice2 vs. Alternative Softwares: The authors assert that the method of polygenic score calculation implemented within PRSice2 generates scores that are comparably predictive to two other methodologies, LDPred and LassoSum. It is my understanding that these methods were developed and are in use precisely because they outperform the method implemented in PRSice in terms of the prediction R-squared for the target phenotype. It would improve the article if the authors could provide some empirical evidence for the claim that their software delivers polygenic scores of comparable accuracy to other methods. For example, comparison of PRSice2 scores to scores generated from LDPred and lassosum for a set of traits would be helpful. I like the choices of height and BMI. But it might also be sensible to consider a trait for which existing GWAS are smaller/ polygenic predictions are less accurate, e.g. depression.<br><br>>> Please see above response<br><br>Re Imputed Genotype Probabilities vs. Allele Counts: The authors helpfully report that PRSice2 scores computed with imputed data can improve prediction accuracy by about 1 percentage point for height and BMI as compared to scores computed with genotyped-only data. It would be helpful to add an element to this analysis. As I understand it, the authors are comparing a genotyped-SNP-only polygenic score computed from allele counts to an imputed-SNP polygenic score computed from genotype probabilities. But these are not the only two possibilities. In much polygenic score analysis, imputed SNP probabilities are converted to discrete genotypes using a threshold (e.g. probability=0.9) to determine whether a given genotype can be assigned to the SNP. Since this is common practice in the field, it seems to me that it would be helpful to include this approach in the comparison.<br><br>>> Please see above response<br><br>Finally, I have one small quibble about language:<br><br>In the introduction, the authors assert that polygenic scores have proven clinical utility. This is a bit of an overstatement. I think we can say that "provocative new data suggest the potential for polygenic scores to be useful in clinical settings" or something similar. The recent papers referenced by the authors are compelling. But the term clinical utility has a specific meaning - that application of a tool improves patient outcomes (e.g. see Torkamani et al. 2018 Nat Rev Genet). We are a long way off from that. Instead, the evidence we have supports an argument for the clinical validity of extreme polygenic-scores values for assessing disease risk.<br><br>>> We thank the reviewer for highlighting this and we entirely agree, that as worded, this could have led readers to a conclusion that we do not agree with ourselves (ie. we also believe that PRS are a long way off clinical utility at the individual-level). We have now changed the introduction as follows (note mention of ‘stratified medicine’ in the revised version, as opposed to personalized medicine):<br><br>“Polygenic Risk Score (PRS) analyses are beginning to play a critical role in biomedical research, being already sufficiently powered to provide scientific insights and with the potential to contribute to stratified medicine in the future [1-9].”
**Additional Information:**	
**Question**	**Response**
Are you submitting this manuscript to a special series or article collection?	No
**Experimental design and statistics**	Yes

Powered by Editorial Manager® and ProduXion Manager® from Aries Systems Corporation

Full details of the experimental design and statistical methods used should be given in the Methods section, as detailed in our Minimum Standards Reporting Checklist. Information essential to interpreting the data presented should be made available in the figure legends.<br><br>Have you included all the information requested in your manuscript?	
**Resources**<br><br>A description of all resources used, including antibodies, cell lines, animals and software tools, with enough information to allow them to be uniquely identified, should be included in the Methods section. Authors are strongly encouraged to cite Research Resource Identifiers (RRIDs) for antibodies, model organisms and tools, where possible.<br><br>Have you included the information requested as detailed in our Minimum Standards Reporting Checklist?	Yes
**Availability of data and materials**<br><br>All datasets and code on which the conclusions of the paper rely must be either included in your submission or deposited in publicly available repositories (where available and ethically appropriate), referencing such data using a unique identifier in the references and in the “Availability of Data and Materials” section of your manuscript.<br><br>Have you have met the above requirement as detailed in our Minimum Standards Reporting Checklist?	Yes

Manuscript

Click here to access/download;Manuscript;20190313-PRSice2-reply.docx

Click here to view linked References

PRSice-2: Polygenic Risk Score Software for Large-Scale Data

Shing Wan Choi[1]* and Paul O'Reilly[1,2]*

1 MRC Social, Genetic and Developmental Psychiatry Centre, Institute of Psychiatry, Psychology and Neuroscience, King's College London, London, United Kingdom

2 Icahn School of Medicine, Mount Sinai, New York City

**To whom correspondence should be addressed.*

Abstract

Background: Polygenic Risk Score (PRS) analyses have become an integral part of biomedical research, exploited to gain insights into shared aetiology among traits, to control for genomic profile in experimental studies, and to strengthen causal inference, among a range of applications. Substantial efforts are now devoted to biobank projects to collect large genetic and phenotypic data, providing unprecedented opportunity for genetic discovery and applications. To process the large-scale data provided by such biobank resources, highly efficient and scalable methods and software are required.

Method: Here we introduce PRSice-2, an efficient and scalable software for automating and simplifying polygenic risk score analyses on large-scale data. PRSice-2 handles both genotyped and imputed data, provides empirical association *P*-values free from inflation due to overfitting, supports different inheritance models and can evaluate multiple continuous and binary target traits

simultaneously. We demonstrate that PRSice-2 is dramatically faster and more memory-efficient than PRSice and alternative polygenic score software, LDpred and lassosum, while having comparable predictive power. This combination of efficiency and power will be increasingly important as data sizes grow and as the applications of PRS become more sophisticated; for example, when incorporated into high-dimensional or gene-set based analyses.

Conclusion: PRSice-2 is written in C++, with an R script for plotting, and is freely available for download from http://PRSice.info

Keywords: Polygenic Risk Score, GWAS, Imputation

Polygenic Risk Score (PRS) analyses are beginning to play a critical role in biomedical research, being already sufficiently powered to provide scientific insights and with the potential to contribute to stratified medicine in the future [1–9]. The increasing availability of genetic data from regional and national biobank projects [10–12] have allowed more powerful PRS to be calculated. However, the calculation of PRS, which involves parameter optimization [13–16], can be a computationally intensive process, especially for large datasets and when multiple analyses are conducted.

To fully utilize the power of large datasets and to facilitate future method and application developments, at scale, we have performed a major overhaul of our original PRSice software [13], to produce PRSice-2. All code has been re-written in C++ and code from PLINK-1.9 [17] has been incorporated to optimize computation. As a result of the consistent language and switch to objected-oriented code, different analytical components of the code can communicate directly,

without, for example, the generation of intermediate files, such as those containing PRS corresponding to each *P*-value threshold, or post-processed genotype files. This has generated a substantial speed-up, a lower processing burden and a reduction in disk space requirement in PRSice-2. In addition, a separate plotting script was implemented in R. Separate tasks are organized into functions and are, thus, more amenable to tailored extensions by users. Finally, a range of user-options were incorporated into PRSice-2 to increase flexibility and improve usability.

Features of PRSice-2

PRSice-2 utilizes the same standard approach to PRS calculation as PRSice, involving clumping Single Nucleotide Polymorphisms (SNPs) (thinning SNPs according to linkage disequilibrium and *P*-value) and then performing *P*-value thresholding, known as the "C+T" method [14], and retains the majority of the features of its predecessor [13], including automatic strand flipping, clumping [18], and calculation and evaluation of PRS under few ('fastscore') or many ('high-resolution scoring') *P*-value thresholds.

When compared to PRSice, PRSice-2 streamlines the entire PRS analysis pipeline without generating intermediate files, and performs all the main computations in C++, leading to a drastic speed-up in runtime and reduction in memory burden (see Supplementary Figure 1). Extraction and exclusion of samples and SNPs are also implemented, allowing PRS analysis to be performed directly on a subset of the input data without performing pre-filtering.

Briefly, the main features of PRSice-2 are:

1. Handles large-scale PRS analyses of both genotyped and imputed data
2. Computes empirical association *P*-values to account for over-fitting
3. Can perform PRS analyses on a large number of target phenotypes simultaneously
4. Provides several options for imputing missing genotypes
5. Allows calculation of PRS based on different inheritance models, including additive, dominant, recessive and heterozygous models
6. Automatically generates dummy variables for categorical covariates
7. Can perform regression to estimate relative effect/risk corresponding to samples in user-defined stratum of the population. Can output quantile and strata plots
8. Amenable to user extensions, such as relating to input data format, regression modelling and output

Handling of Imputed data

Genotypes are typically represented as the discrete counts of the minor or effect allele (0, 1 or 2), for single nucleotide polymorphisms (SNPs), in each individual. Genotypes not included in the genotyping chip can, potentially, be imputed and are usually either recorded as a set of three probabilities corresponding to the probability of each of the possible genotypes [19], or based on these, as the expected genotype (a real number between 0 and 2 known as the "dosage") [19] or as the "best-guess" (most probable) genotype. While any of these data formats can be exploited in PRS analyses, the most common approach is to use the "best-guess" genotype for each individual. However, this approach does not account for the uncertainty in the imputed genotype.

Currently, most PRS software only support input of the genotyped format. Therefore, users need to generate a large intermediate file containing the best-guess genotypes and discard any information related to imputation uncertainty. To reduce the storage space requirement, and to incorporate imputation uncertainty into PRS analyses, PRSice-2 implements support for the BGEN imputation format. PRSice-2 can directly process the BGEN imputed format and either convert to best-guess genotypes or dosages when calculating the PRS, without generating a large intermediate file. While PRS based on best-guess genotypes are calculated as for genotyped input, dosage based PRS are calculated as

$$PRS = \left(\sum_{i}^{m} \beta_i \left(\sum_{j=0}^{2} \omega_{ij} \times j \right) \right) \quad (1)$$

where ω_{ij} is the probability of observing genotype j , where $j \in \{0,1,2\}$, for the ith SNP, m is the number of SNPs and β_i is the effect size of the ith SNP estimated from the relevant base GWAS data.

The ability to perform PRS analyses directly on imputed data can be particularly useful when the base GWAS and target samples are genotyped on a different platform, as then there can be a small fraction of overlapping SNPs. For example, of the 725,459 post-QC SNPs (see Supplementary Material) in the UK Biobank genotype data [10], only 31% (222,956) of those were found in the GIANT Height and Body Mass Index (BMI) GWAS [20,21]. The use of imputed SNPs increases the number of overlapping SNPs to 2,121,036 SNPs. To assess the gain in power when using imputed vs un-imputed data, we performed PRS analyses on height and BMI using UK Biobank

genotyped and imputed data, with GWAS summary statistics provided by the GIANT consortium [20,21]. Age, sex, UK Biobank genotyping batch, UK Biobank assessment centre and 40 principle components were first regressed out from the phenotype and the standardized residuals were used instead.

We performed a linear regression using PRSice-2, with the UK Biobank data as target sample using the default parameters. When calculating PRS from the "best-guess" genotype, the "best-guess" genotype is defined as the genotype having an imputation probability of 0.9 or above. If there is no such genotype, then the SNP is considered to be missing for the individual. In addition, for the imputed data, we filtered out SNPs with imputation quality score less than 0.8. With height as the outcome and PRS for height as predictor, we observed an increase in phenotypic variance explained (R^2) of the PRS from 0.145 when using genotyped data to 0.152 when using best-guess imputed genotypes, and 0.153 when using dosage data; likewise, the R^2 for BMI increased from 0.0475 when using genotype data to 0.0529 when using best-guess genotypes, and to 0.0535 when using dosage data. These results exemplify the potential gain in predictive power when using dosage data compared to using genotyped or best-guess genotype data. However, given the modest increases in predictive power, users may wish to perform first-pass analyses on genotyped-only data before application to the more computationally intensive imputed data. A further challenge in exploiting imputed data is that there are numerous imputed formats in use in the field. While it is difficult to support all imputed formats, PRSice-2 adopts a modular approach, which allows simple incorporation of supports for additional data formats (eg. vcf) in the future.

Calculation of Empirical *P*-value

All approaches to PRS calculation involve parameter optimisation in generating the final prediction model, and are thus vulnerable to overfitting [14]. The best strategy to avoid overfitting is to evaluate performance in an independent validation sample, but such a sample is not always available. Alternatively, if the primary aim is to assess evidence for an association to test a hypothesis, then we can calculate an empirical *P*-value corresponding to the association of the optimized PRS, with the Type 1 error rate controlled [13].

In PRSice-2, to obtain the empirical *P*-value, the target trait values are permuted across the sample of individuals *k* times (default = 10,000) and the PRS analysis is repeated on each set of permuted phenotypes. Thus, on each permutation, the "best-fit PRS" is obtained as that most associated with the target trait across the range of *P*-value thresholds considered, and the empirical *P*-value is calculated as:

$$empirical\ P = \frac{\sum_{n=1}^{N} I(P_n < P_o) + 1}{N + 1} \qquad (2)$$

where *N* is the number of permutations performed, *I*(.) is the indicator function, which takes a value of 0 if the "best-fit PRS" of permutation *n* is smaller than the observed *P*-value, P_o, and where pseudo-counts of 1 are added to the numerator and denominator to avoid empirical *P*-values of 0 and reflecting (conservatively) counting the observed trait configuration as one potential null permutation [22]. While the empirical *P*-values for association will be controlled for the Type 1 error rate, since the same process of parameter optimisation is performed explicitly under the null hypothesis, the observed phenotypic variance explained, R^2, remains unadjusted and is affected by overfitting. Therefore, it is imperative to perform out-of-sample prediction, or cross-validation, to

evaluate the predictive accuracy of PRS when using PRSice-2, and ideally the former given the problems of generalisability observed with PRS [14].

Analysis of PRS strata

While PRS on most complex traits presently have limited power to accurately predict risk at the individual-level, which will remain the case for low-moderate heritability traits irrespective of GWAS sample sizes, recent studies have demonstrated that individuals at the tails of PRS distribution can have substantially higher disease risk than those of the general population. Thus, these individuals may provide useful subjects for experimental follow-up, while in clinical settings it could be more efficacious to employ different risk management strategies, in terms of screening or interventions, for example, for individuals with extreme PRS [1–3].

We have implemented a strata analysis feature in PRSice-2 to aid the calculation of relative phenotypic risk of individuals between strata. Briefly, the N individuals of the target sample are first aggregated into M different strata based on their PRS. An N x $(M - 1)$ design matrix is then generated using dummy coding, such that an individual is coded 1 in the column that corresponds to their PRS stratum and whereby a user-defined stratum is the reference group (or the median stratum by default). A linear regression (for quantitative traits) or logistic regression (for binary traits) will then be performed to estimate the phenotypic difference or relative risk, respectively, of each stratum versus the reference. The set of corresponding beta-coefficients (linear) or the odds ratio (logistic), can then be visualized with the strata plot (Figure 1). This allow users to assess whether individuals in the extreme stratum have a substantially higher phenotypic risk when compared to the reference stratum.

Figure 1

Figure 1 Strata plot generated by PRSice-2. The X-axis shows the range of different quantiles (eg. (80,90] corresponds to those individuals with PRS between the 80%-ile – 90%-ile of the population), and the Y-axis shows the odds ratio (OR) when comparing PRS from different quantiles with the reference quantile (here, (40,60]).

Benchmarking

Here we perform a simulation study to compare the performance of PRSice-2 to alternative polygenic score software lassosum [15] and LDpred [16], in terms of runtime, memory usage and predictive power.

Quantitative traits with heritability (h^2) of 0.2, 0.4, 0.6 and 0.8 were simulated with the UK Biobank genotype data (post-QC) as input. Briefly, each quantitative trait was simulated based on the following linear model:

$$Y = X\beta + \varepsilon \qquad (3)$$

where X is the unstandardized genotype matrix corresponding to 385,794 individuals (rows) and 560,173 SNP genotypes (columns). The β vector corresponds to the effect size associated with each SNP, with 100, 1k, 10k, 100k and 560,173 (all SNPs) randomly selected to be causal SNPs with effect size $\beta \sim N(0,1)$, β = 0 otherwise, and ε represents the random error, which follows $\varepsilon \sim N\left(0, \sqrt{\frac{var(X\beta)(1-h^2)}{h^2}}\right)$. To control for batch effects and population structure in the genotype data, a regression of batch, UK Biobank assessment centre and 40 PCs against the simulated trait were performed as follows:

$$Y = Batch + Centre + 40\ PCs + \varepsilon \qquad (4)$$

The standardized residuals were then used as the final simulated trait. 20k samples were randomly selected as the base sample and used to generate the GWAS summary statistics. 100, 1k, 10k and 100k samples independent from the base were then randomly selected as the target sample. PRS analyses were then performed on these base and target data using the latest version of lassosum (v0.4.3), LDpred (v1.0.0) and PRSice 2 (v2.1.8), on servers equipped with two 10 core Intel Haswell E5-2660 v3 @ 2.60GHz and 128GB of RAM. Default parameters of each

program were used. The runtime and memory usage of each program were measured using the Linux *time* command and the predictive power of the methods was assessed according to phenotypic variance explained (R^2). The entire process was repeated 10 times to obtain an estimated distribution of runtime, memory usage and predictive power.

Figure 2 shows the runtime and memory usage of PRSice-2, lassosum and LDpred. Based on these simulation results, PRSice-2 is the most efficient software in all settings (Figure 2a), significantly faster than lassosum (*P* = 3.06e-49, one sided t-test) and LDpred (*P* = 9.06e-86, one sided t-test). Specifically, PRSice-2 can complete the full PRS analysis on 100k samples within 8 minutes (Supplementary Table 1), which is 78x faster than the 9 hours 21 minutes required by lassosum, and 109x faster than the 13 hours 7 minutes required by LDpred. Likewise, PRSice-2 requires significantly less memory (Figure 2b) than lassosum (*P* = 1.13e-150, one sided t-test) and LDpred (*P* = 1.21e-139, one sided t-test), requiring less than 500MB of memory for 100k samples, as opposed to 11.6GB required by lassosum and 38.1 GB required by LDpred (Supplementary Table 2). Likewise, PRSice-2 outperforms PRSice-v1.25, requiring 200x less time and 7x less memory for a target sample size of 10k (similar memory for small target samples. See Supplementary Figure 1, Supplementary Tables 1,2 for details). As data size grows, or when more sophisticated PRS analyses are performed at scale [5,23], these gains in computational efficiency could become even more important.

Figure 2a	Figure 2b

Figure 2 Performance of the three PRS software on simulated data. a) Average run time (in minutes) required to complete the entire analysis, across 10 repeats, when applied to different sizes of target sample. b) Average memory (in GB) required for the different software to process the different sizes of target sample.

Figure 3 shows the predictive power of PRSice-2 when compared to lassosum and LDpred for quantitative traits with heritability of 0.6 and target sample size of 10k (see Supplementary Figure 2 for comparisons across all settings). Consistent with previous findings [15,24,25], PRSice-2 has comparable predictive power to lassosum and LDpred, generating PRS with predictive power higher than that of LDpred but not as high as lassosum. These results are

inherently dependent on modelling assumptions and we provide these only as an approximate guide of performance in settings that match our assumptions. We provide our simulation code (https://github.com/choishingwan/PRSice-paper-script) for others to inspect and repeat our analyses.

While PRS generated by PRSice-2 do not appear to fully optimize predictive accuracy, the simple approach and typically fewer SNPs exploited allows for easier interpretation of the results compared to methods that use all SNPs [26]. Moreover, the efficiency and predictive power of PRSice-2 makes it an ideal tool to perform PRS analyses at scale.

Figure 3

Figure 3 Predictive accuracy of the three PRS software for a simulated trait with heritability h2=0.6 and target sample size of 10k. The Y-axis represents the trait variance explained (R^2) by the PRS generated from each software, while the X-axis corresponds to the number of causal SNPs for the simulated trait. Full results of the comparison study are shown in Supplementary Figure 2.

Discussion

We have introduced PRSice-2, a software for the automation of polygenic risk score (PRS) analyses applied to large-scale genotype-phenotype data. Our results demonstrate that PRSice-2 is the most efficient among some of the leading PRS software, outperforming lassosum [15] and LDpred [16]. As data sizes increase and more complicated PRS analyses, such as multi-trait or gene-set based PRS analyses, become common, the efficiency advantages of PRSice-2 will become increasingly important.

Over-fitting is a concern for all approaches to PRS analyses [14]. To control for the Type 1 error rate caused by over-fitting when exploiting PRS for hypothesis testing, PRSice-2 implements the calculation of empirical *P*-values.

PRSice-2 implements a standard approach for performing PRS analyses. For PRS analyses performed in family data or across diverse populations, for instance, results should be interpreted carefully [14] and extensions of the standard PRS approach or alternatives may be required [14,27–29] to generate more informative results.

Availability and requirements

**Project Name**	PRSice-2
**Project home page**	http://prsice.info
**Operating systems (pre-compiled versions)**	Linux (64-bit)<br>OS X (64-bit Intel)<br>Windows (64-bit)
**Programming language**	C++, R (version 3.2.3+)
**Other requirements (when recompiling)**	GCC version 4.8+, zlib
**License**	GNU General Public License version 3.0 (GPLv3)
**Any restrictions to use by non-academics**	None
**RRID**	SCR_017057

Declarations

Acknowledgements

We thank the participants in the UK Biobank and the scientists involved in the construction of this resource. This research has been conducted using the UK Biobank Resource under application 18177 (Dr O'Reilly). We thank Hei Man Wu for providing critical feedback regarding this manuscript and for test running the software. We thank Jonathan Coleman and Kylie Glanville for the management of the UK Biobank resource at King's College London, and we thank Jack Euesden for his work on PRSice, which forms the basis of the current software. We thank Christopher Hübel, Eva Krapohl, Kirstin Purves, Jessye Maxwell, Saskia Hagenaars and Yunfeng Ruan for their help in test running the software. PFO receives funding from the UK Medical Research Council (MR/N015746/1). SWC is funded from the UK Medical Research Council (MR/N015746/1). This report represents independent research (part)-funded by the National Institute for Health Research (NIHR) Biomedical Research Centre at South London and Maudsley NHS Foundation Trust and King's College London. The views expressed are those of the authors and not necessarily those of the NHS, the NIHR, or the Department of Health.

Competing Interests

The authors declare that they have no competing interests

Authors' contributions

SWC and PFO designed the software. SWC implemented the software and drafted the manuscript. PFO provided critical feedback regarding the manuscript and the software development. All authors read and approved the final manuscript.

References

1. Mavaddat N, Pharoah PDP, Michailidou K, Tyrer J, Brook MN, Bolla MK, et al. Prediction of Breast Cancer Risk Based on Profiling With Common Genetic Variants. JNCI J Natl Cancer Inst [Internet]. 2015 [cited 2017 Jun 13];107. Available from: https://academic.oup.com/jnci/article/107/5/djv036/891009/Prediction-of-Breast-Cancer-Risk-Based-on

2. Kuchenbaecker KB, McGuffog L, Barrowdale D, Lee A, Soucy P, Healey S, et al. Evaluation of Polygenic Risk Scores for Breast and Ovarian Cancer Risk Prediction in BRCA1 and BRCA2 Mutation Carriers. JNCI J Natl Cancer Inst [Internet]. 2017 [cited 2018 Sep 26];109. Available from: https://www.ncbi.nlm.nih.gov/pmc/articles/PMC5408990/

3. Natarajan P, Young R, Stitziel NO, Padmanabhan S, Baber U, Mehran R, et al. Polygenic Risk Score Identifies Subgroup with Higher Burden of Atherosclerosis and Greater Relative Benefit from Statin Therapy in the Primary Prevention Setting. Circulation. 2017;CIRCULATIONAHA.116.024436.

4. Udler MS, Kim J, Grotthuss M von, Bonas-Guarch S, Mercader JM, Cole JB, et al. Clustering of Type 2 Diabetes Genetic Loci by Multi-Trait Associations Identifies Disease Mechanisms and Subtypes. bioRxiv. 2018;319509.

5. Krapohl E, Euesden J, Zabaneh D, Pingault J-B, Rimfeld K, von Stumm S, et al. Phenome-wide analysis of genome-wide polygenic scores. Mol Psychiatry. 2016;21:1188–93.

6. Krapohl E, Patel H, Newhouse S, Curtis CJ, Stumm S von, Dale PS, et al. Multi-polygenic score approach to trait prediction. Mol Psychiatry. 2018;23:1368–74.

7. Selzam S, Krapohl E, von Stumm S, O'Reilly PF, Rimfeld K, Kovas Y, et al. Predicting educational achievement from DNA. Mol Psychiatry. 2017;22:267–72.

8. Selzam S, Dale PS, Wagner RK, DeFries JC, Cederlöf M, O'Reilly PF, et al. Genome-Wide Polygenic Scores Predict Reading Performance Throughout the School Years. Sci Stud Read. 2017;21:334–49.

9. Du Rietz E, Coleman J, Glanville K, Choi SW, O'Reilly PF, Kuntsi J. Association of Polygenic Risk for Attention-Deficit/Hyperactivity Disorder With Co-occurring Traits and Disorders. Biol Psychiatry Cogn Neurosci Neuroimaging. 2018;3:635–43.

10. Sudlow C, Gallacher J, Allen N, Beral V, Burton P, Danesh J, et al. UK Biobank: An Open Access Resource for Identifying the Causes of a Wide Range of Complex Diseases of Middle and Old Age. PLOS Med. 2015;12:e1001779.

11. Danciu I, Cowan JD, Basford M, Wang X, Saip A, Osgood S, et al. Secondary use of clinical data: The Vanderbilt approach. J Biomed Inform. 2014;52:28–35.

12. Kaiser J. NIH’s 1-million-volunteer precision medicine study announces first pilot projects. Science [Internet]. 2016 [cited 2018 Nov 15]; Available from: https://www.sciencemag.org/news/2016/02/nih-s-1-million-volunteer-precision-medicine-study-announces-first-pilot-projects

13. Euesden J, Lewis CM, O’Reilly PF. PRSice: Polygenic Risk Score software. Bioinformatics. 2015;31:1466–8.

14. Choi SW, Mak TSH, O’Reilly P. A guide to performing Polygenic Risk Score analyses. bioRxiv. 2018;416545.

15. Mak TSH, Porsch RM, Choi SW, Zhou X, Sham PC. Polygenic scores via penalized regression on summary statistics. Genet Epidemiol. 2017;41:469–80.

16. Vilhjálmsson BJ, Yang J, Finucane HK, Gusev A, Lindström S, Ripke S, et al. Modeling Linkage Disequilibrium Increases Accuracy of Polygenic Risk Scores. Am J Hum Genet. 2015;97:576–92.

17. Chang CC, Chow CC, Tellier LC, Vattikuti S, Purcell SM, Lee JJ. Second-generation PLINK: rising to the challenge of larger and richer datasets. GigaScience. 2015;4:7.

18. Wray NR, Lee SH, Mehta D, Vinkhuyzen AAE, Dudbridge F, Middeldorp CM. Research review: Polygenic methods and their application to psychiatric traits. J Child Psychol Psychiatry. 2014;55:1068–87.

19. Li Y, Willer C, Sanna S, Abecasis G. Genotype Imputation. Annu Rev Genomics Hum Genet. 2009;10:387–406.

20. Wood AR, Esko T, Yang J, Vedantam S, Pers TH, Gustafsson S, et al. Defining the role of common variation in the genomic and biological architecture of adult human height. Nat Genet. 2014;46:1173–86.

21. Locke AE, Kahali B, Berndt SI, Justice AE, Pers TH, Day FR, et al. Genetic studies of body mass index yield new insights for obesity biology. Nature. 2015;518:197–206.

22. North BV, Curtis D, Sham PC. A Note on the Calculation of Empirical P Values from Monte Carlo Procedures. Am J Hum Genet. 2002;71:439–41.

23. Hagenaars SP, Harris SE, Davies G, Hill WD, Liewald DCM, Ritchie SJ, et al. Shared genetic aetiology between cognitive functions and physical and mental health in UK Biobank (N=112 151) and 24 GWAS consortia. Mol Psychiatry. 2016;21:1624–32.

24. Allegrini A, Selzam S, Rimfeld K, Stumm S von, Pingault J-B, Plomin R. Genomic prediction of cognitive traits in childhood and adolescence. bioRxiv. 2018;418210.

25. Ge T, Chen C-Y, Ni Y, Feng Y-CA, Smoller JW. Polygenic Prediction via Bayesian Regression and Continuous Shrinkage Priors. bioRxiv. 2018;416859.

26. Janssens ACJW, Joyner MJ. Polygenic Risk Scores That Predict Common Diseases Using Millions of Single Nucleotide Polymorphisms: Is More, Better? Clin Chem. 2019;clinchem.2018.296103.

27. Duncan L, Shen H, Gelaye B, Ressler K, Feldman M, Peterson R, et al. Analysis of Polygenic Score Usage and Performance across Diverse Human Populations. bioRxiv. 2018;398396.

28. Márquez- Luna C, Loh P-R, Price AL. Multiethnic polygenic risk scores improve risk prediction in diverse populations. Genet Epidemiol. 2017;41:811–23.

29. Martin AR, Gignoux CR, Walters RK, Wojcik GL, Neale BM, Gravel S, et al. Human Demographic History Impacts Genetic Risk Prediction across Diverse Populations. Am J Hum Genet. 2017;100:635–49.

Figure 1

Click here to access/download;Figure;Figure 1.png

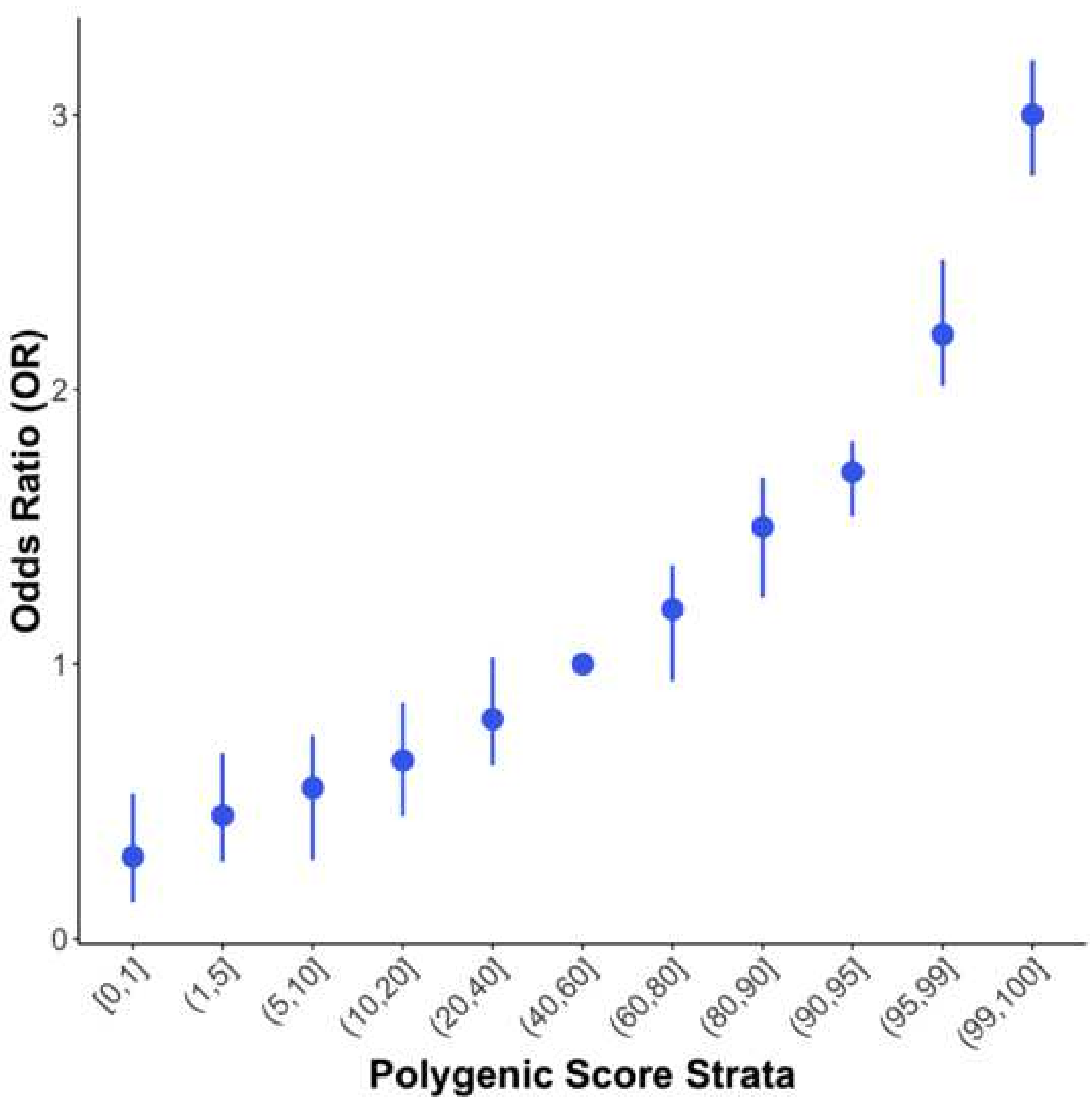

Figure 2a

Click here to access/download;Figure;Figure 2a.png

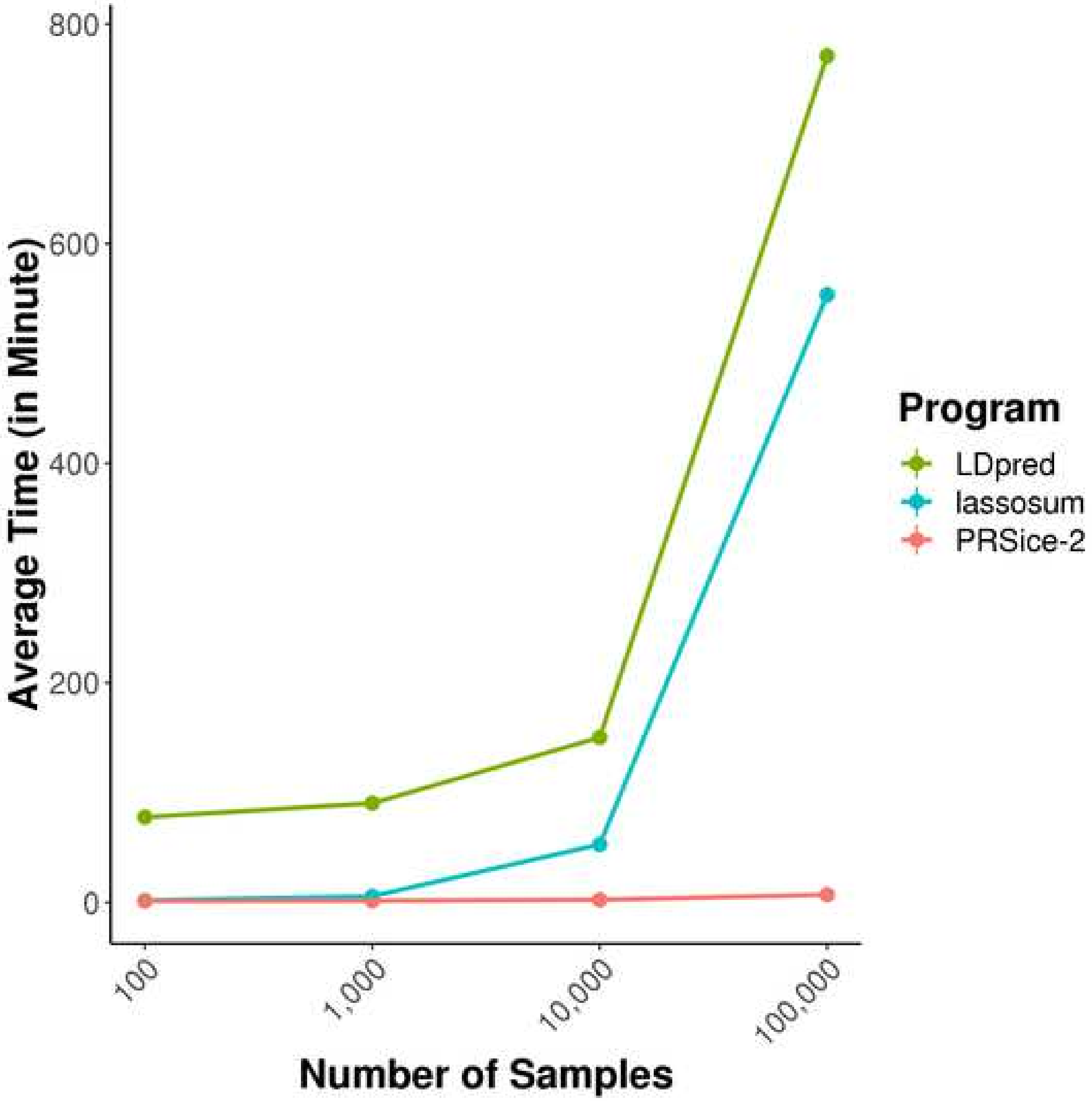

 Click here to access/download;Figure;Figure 2b.png

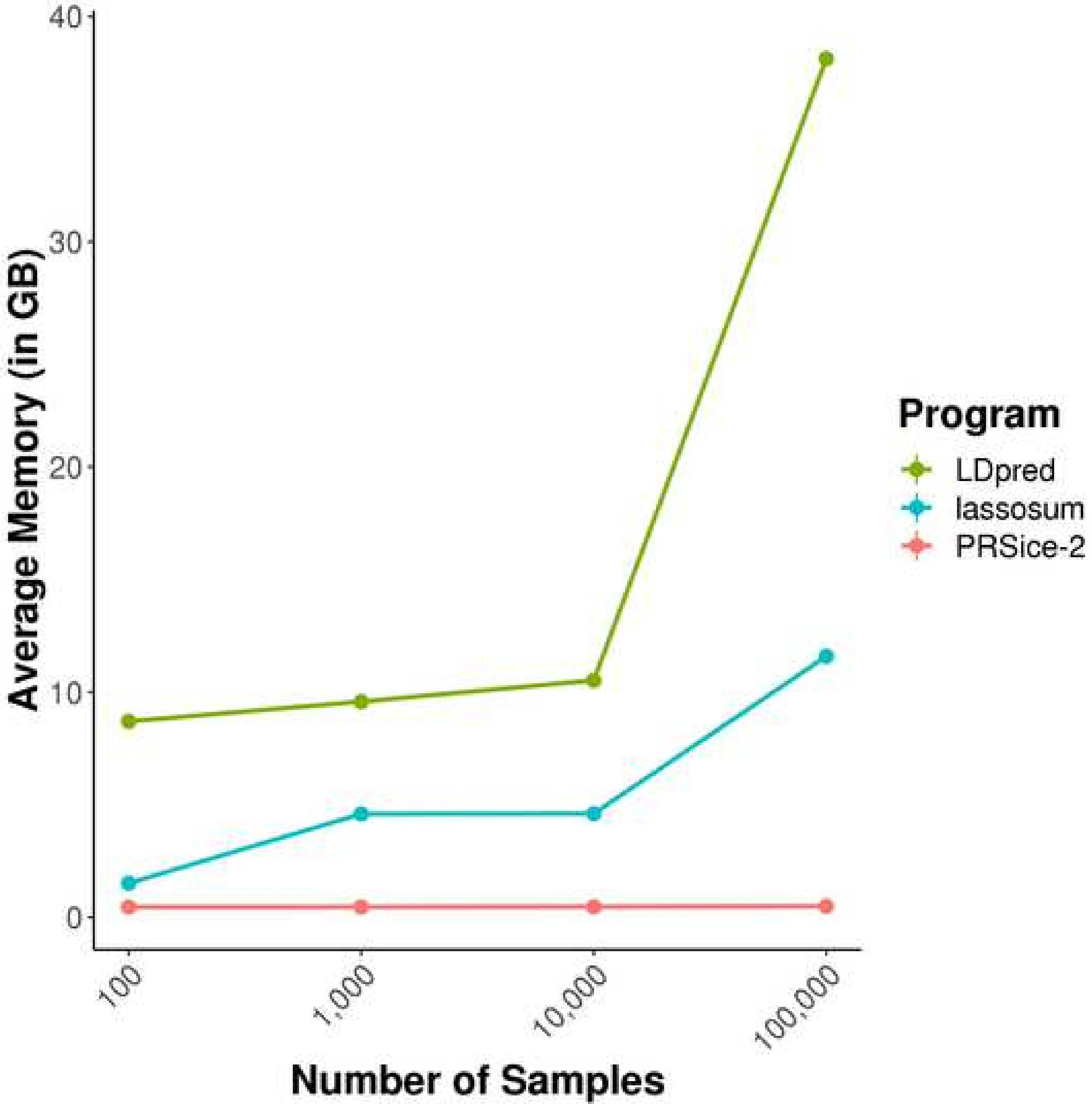

 Click here to access/download;Figure;Figure 3.png

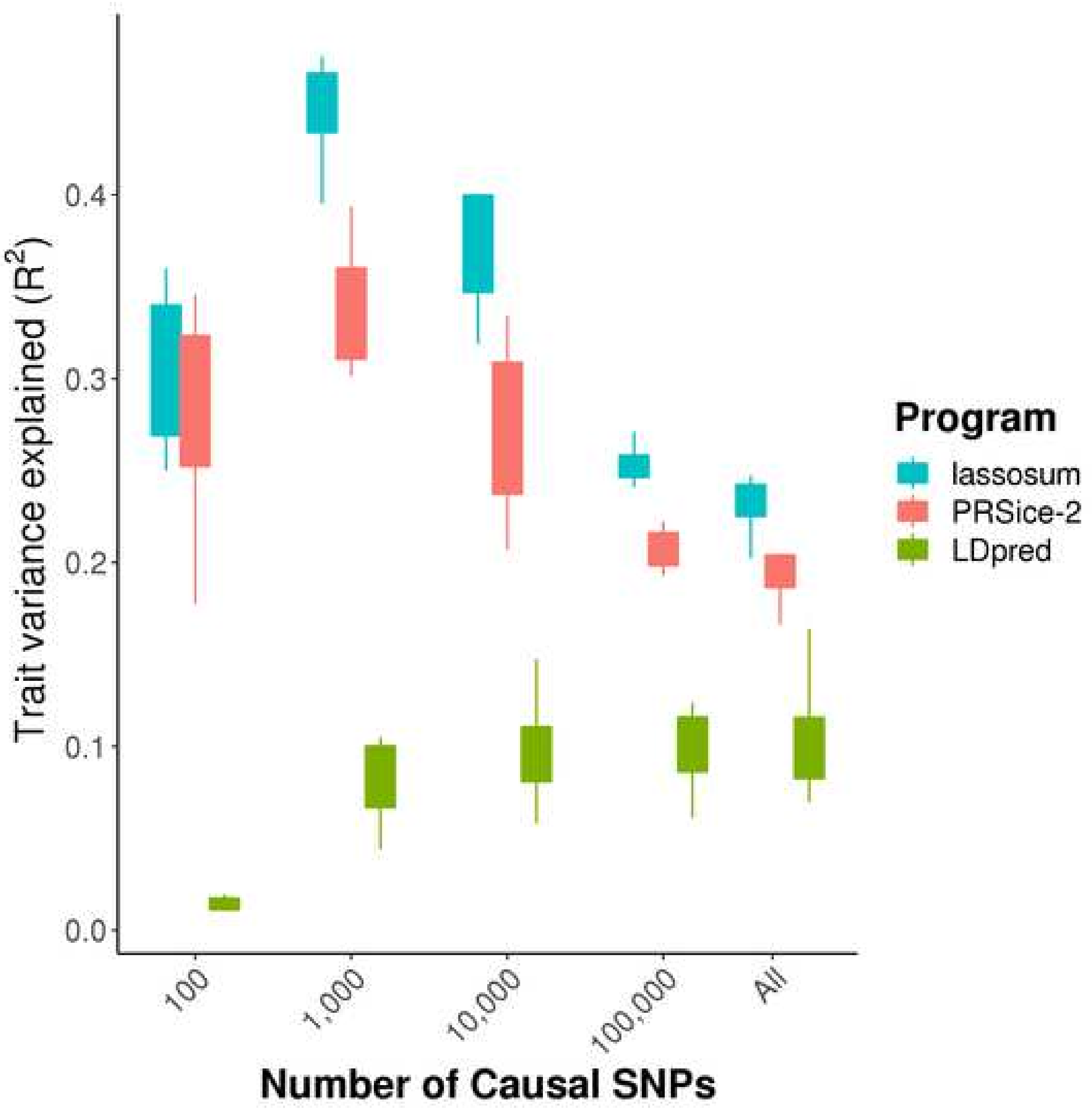

Click here to access/download
Supplementary Material
20190313-PRSice2 Supplementary.docx

Personal Cover
Click here to access/download;Personal Cover;20190313-CoverLetter_PRSice2.docx

MRC Social, Genetic &
Developmental Psychiatry Centre
Director **Francesca Happé**

16 De Crespigny Park
Denmark Hill
London SE5 8AF

Dr Shing Wan Choi
Email: shing_wan.choi@kcl.ac.uk
Telephone: +44 (0)7729246486

PRSice-2: Polygenic Risk Score Analysis Software for Large-Scale Data *(For submission as a Technical Note)*

13th March 2019

Dear Editor

Thank you for the letter dated 16th Jan 2019. The reviewers' comments are insightful and have helped to guide us to improve our paper. Enclosed is the latest version of our manuscript "PRSice-2: Polygenic Risk Score Analysis Software for Large-Scale Data" (GIGA-D-18-00468).

In this revision, we have included a comparison of the performance of polygenic risk scores computed using genotyped data only, using imputed SNP probabilities and from best-guess genotypes in the UKBB genotype data. We have also performed a comprehensive simulation study to compare the runtime, memory burden and predictive accuracy of PRSice-2 to leading alternatives lassosum and LDpred. We also included runtime/memory comparisons between PRSice-2 and PRSice-v1.25 and revised the main text in a number of areas according to reviewer suggestions.

We believe that our updated manuscript addresses all the concerns raised by the reviewers and is a much-improved piece of work for it. We hope that our paper is now suitable for publication in GigaScience as a 'Technical Note'. We look forward to hearing back from you on this.

Shing Wan Choi (cc'ed Paul F. O'Reilly)
