# Supplementary material for: PRSice-2: Polygenic Risk Score software for biobank-scale data
Source: Gigascience. 2019 Jul 15;8(7):giz082. doi: 10.1093/gigascience/giz082 (PMC6629542; doi:10.1093/gigascience/giz082)
Supplement: giz082_GIGA-D-18-00468_Revision_2 [file giz082_giga-d-18-00468_revision_2.pdf]

GigaScience

PRSice-2: Polygenic Risk Score software for biobank-scale data

--Manuscript Draft--

**Manuscript Number:**	GIGA-D-18-00468R2
**Full Title:**	PRSice-2: Polygenic Risk Score software for biobank-scale data
**Article Type:**	Technical Note
**Funding Information:**	UK Medical Research Council (MR/N015746/1) — Dr Shing Wan Choi<br>Dr Paul F O'Reilly
**Abstract:**	Background<br><br>Polygenic Risk Score (PRS) analyses have become an integral part of biomedical research, exploited to gain insights into shared aetiology among traits, to control for genomic profile in experimental studies, and to strengthen causal inference, among a range of applications. Substantial efforts are now devoted to biobank projects to collect large genetic and phenotypic data, providing unprecedented opportunity for genetic discovery and applications. To process the large-scale data provided by such biobank resources, highly efficient and scalable methods and software are required.<br><br>Method<br><br>Here we introduce PRSice-2, an efficient and scalable software for automating and simplifying polygenic risk score analyses on large-scale data. PRSice-2 handles both genotyped and imputed data, provides empirical association P-values free from inflation due to overfitting, supports different inheritance models and can evaluate multiple continuous and binary target traits simultaneously. We demonstrate that PRSice-2 is dramatically faster and more memory-efficient than PRSice-1 and alternative polygenic score software, LDpred and lassosum, while having comparable predictive power. This combination of efficiency and power will be increasingly important as data sizes grow and as the applications of PRS become more sophisticated; for example, when incorporated into high-dimensional or gene-set based analyses.<br><br>Conclusion<br><br>PRSice-2 is written in C++, with an R script for plotting, and is freely available for download from http://PRSice.info
**Corresponding Author:**	Shing Wan Choi<br>King's College London<br>London, UNITED KINGDOM
**Corresponding Author Secondary Information:**	
**Corresponding Author's Institution:**	King's College London
**Corresponding Author's Secondary Institution:**	
**First Author:**	Shing Wan Choi
**First Author Secondary Information:**	
**Order of Authors:**	Shing Wan Choi<br>Paul F O'Reilly
**Order of Authors Secondary Information:**	
**Response to Reviewers:**	We contacted the first author of LDpred, Dr Bjarni Vilhjalmsson. He informed us that LDpred can become sensitive to small deviations in LD estimates when there are large sample sizes in application to a trait with high heritability. We also noted that there is a

Powered by Editorial Manager® and ProduXion Manager® from Aries Systems Corporation

	new version of LDpred (v1.0.6) now available. Repeating our analyses using a smaller base sample size of 50000, and using the latest version of LDpred, we noted that the performance of LDpred substantially improved. As a result of this, we repeated our entire analyses using the latest versions of PRSice-2 and LDpred and have updated our results accordingly. The overall results remain qualitatively unchanged: PRSice-2 is still markedly the fastest PRS program (more so than previously) and it has comparable power to lassosum and LDpred, with predictive power higher than LDpred and lower than lassosum.
**Additional Information:**	
**Question**	**Response**
Are you submitting this manuscript to a special series or article collection?	No
**Experimental design and statistics**<br><br>Full details of the experimental design and statistical methods used should be given in the Methods section, as detailed in our Minimum Standards Reporting Checklist. Information essential to interpreting the data presented should be made available in the figure legends.<br><br>Have you included all the information requested in your manuscript?	Yes
**Resources**<br><br>A description of all resources used, including antibodies, cell lines, animals and software tools, with enough information to allow them to be uniquely identified, should be included in the Methods section. Authors are strongly encouraged to cite Research Resource Identifiers (RRIDs) for antibodies, model organisms and tools, where possible.<br><br>Have you included the information requested as detailed in our Minimum Standards Reporting Checklist?	Yes
**Availability of data and materials**<br><br>All datasets and code on which the conclusions of the paper rely must be either included in your submission or	Yes

deposited in publicly available repositories (where available and ethically appropriate), referencing such data using a unique identifier in the references and in the “Availability of Data and Materials” section of your manuscript.<br><br>Have you have met the above requirement as detailed in our Minimum Standards Reporting Checklist?	

Manuscript with DOI
Click here to view linked References
Click here to access/download;Manuscript;20190604-PRSice2-reply NN PFO with DOI.docx

PRSice-2: Polygenic Risk Score software for biobank-scale data

Shing Wan Choi[1,2]* and Paul O'Reilly[1,2]*

1 MRC Social, Genetic and Developmental Psychiatry Centre, Institute of Psychiatry, Psychology and Neuroscience, King's College London, London, United Kingdom

2 Department of Genetics and Genomic Sciences, Icahn School of Medicine, Mount Sinai, New York City

*To whom correspondence should be addressed.

Abstract

Background: Polygenic Risk Score (PRS) analyses have become an integral part of biomedical research, exploited to gain insights into shared aetiology among traits, to control for genomic profile in experimental studies, and to strengthen causal inference, among a range of applications. Substantial efforts are now devoted to biobank projects to collect large genetic and phenotypic data, providing unprecedented opportunity for genetic discovery and applications. To process the large-scale data provided by such biobank resources, highly efficient and scalable methods and software are required.

Method: Here we introduce PRSice-2, an efficient and scalable software for automating and simplifying polygenic risk score analyses on large-scale data. PRSice-2 handles both genotyped and imputed data, provides empirical association *P*-values free from inflation due to overfitting, supports different inheritance models and can evaluate multiple continuous and binary target traits simultaneously. We demonstrate that PRSice-2 is dramatically faster and more memory-efficient

than PRSice-1 and alternative polygenic score software, LDpred and lassosum, while having comparable predictive power. This combination of efficiency and power will be increasingly important as data sizes grow and as the applications of PRS become more sophisticated; for example, when incorporated into high-dimensional or gene-set based analyses.

Conclusion: PRSice-2 is written in C++, with an R script for plotting, and is freely available for download from http://PRSice.info

Keywords: Polygenic Risk Score, GWAS, Imputation

Polygenic Risk Score (PRS) analyses are beginning to play a critical role in biomedical research, being already sufficiently powered to provide scientific insights and with the potential to contribute to stratified medicine in the future [1–9]. The increasing availability of genetic data from regional and national biobank projects [10–12] have allowed more powerful PRS to be calculated. However, the calculation of PRS, which involves parameter optimization [13–16], can be a computationally intensive process, especially for large datasets and when multiple analyses are conducted.

To fully utilize the power of large datasets and to facilitate future method and application developments, at scale, we have performed a major overhaul of our original PRSice software [13], to produce PRSice-2. All code has been re-written in C++ and code from PLINK-1.9 [17] has been incorporated to optimize computation. As a result of the consistent language and switch to objected-oriented code, different analytical components of the code can communicate directly, without, for example, the generation of intermediate files, such as those containing PRS corresponding to each *P*-value threshold, or post-processed genotype files. This has generated a substantial speed-up, a lower processing burden and a reduction in disk space requirement in

PRSice-2. In addition, a separate plotting script is implemented in R. Separate tasks are organized into functions and are, thus, more amenable to tailored extensions by users. Finally, a range of user-options are incorporated into PRSice-2 to increase flexibility and improve usability.

Features of PRSice-2

PRSice-2 utilizes the same standard approach to PRS calculation as PRSice, involving clumping Single Nucleotide Polymorphisms (SNPs) (thinning SNPs according to linkage disequilibrium and *P*-value) and then performing *P*-value thresholding, known as the “C+T” method [14], and retains the majority of the features of its predecessor [13], including automatic strand flipping, clumping [18], and calculation and evaluation of PRS under few (‘fastscore’) or many (‘high-resolution scoring’) *P*-value thresholds.

When compared to PRSice, PRSice-2 streamlines the entire PRS analysis pipeline without generating intermediate files, and performs all the main computations in C++, leading to a drastic speed-up in runtime and reduction in memory burden (see Supplementary Figure 1). Extraction and exclusion of samples and SNPs are also implemented, allowing PRS analysis to be performed directly on a subset of the input data without performing pre-filtering.

Briefly, the main features of PRSice-2 are:

1. Handles large-scale PRS analyses of both genotyped and imputed data
2. Computes empirical association *P*-values to account for over-fitting
3. Can perform PRS analyses on a large number of target phenotypes simultaneously
4. Provides several options for imputing missing genotypes

5. Allows calculation of PRS based on different inheritance models, including additive, dominant, recessive and heterozygous models
6. Automatically generates dummy variables for categorical covariates
7. Can perform regression to estimate relative effect/risk corresponding to samples in user-defined stratum of the population. Can output quantile and strata plots
8. Amenable to user extensions, such as relating to input data format, regression modelling and output

Handling of Imputed data

Genotypes are typically represented as the discrete counts of the minor or effect allele (0, 1 or 2), for single nucleotide polymorphisms (SNPs), in each individual. Genotypes not included in the genotyping chip can, potentially, be imputed and are usually either recorded as a set of three probabilities corresponding to the probability of each of the possible genotypes [19], or based on these, as the expected genotype (a real number between 0 and 2 known as the "dosage") [19] or as the "best-guess" (most probable) genotype. While any of these data formats can be exploited in PRS analyses, the most common approach is to use the "best-guess" genotype for each individual. However, this approach does not account for the uncertainty in the imputed genotype.

Currently, most PRS software only support input of the genotyped format. Therefore, users need to generate a large intermediate file containing the best-guess genotypes and discard any information related to imputation uncertainty. To reduce the storage space requirement, and to incorporate imputation uncertainty into PRS analyses, PRSice-2 implements support for the BGEN imputation format. PRSice-2 can directly process the BGEN imputed format and either convert to

best-guess genotypes or dosages when calculating the PRS, without generating a large intermediate file. While PRS based on best-guess genotypes are calculated as for genotyped input, dosage based PRS are calculated as

$$PRS = \left(\sum_{i}^{m} \beta_i \left(\sum_{j=0}^{2} \omega_{ij} \times j \right) \right) \tag{1}$$

where ω_{ij} is the probability of observing genotype j , where $j \in \{0,1,2\}$, for the ith SNP, m is the number of SNPs and β_i is the effect size of the ith SNP estimated from the relevant base GWAS data.

The ability to perform PRS analyses directly on imputed data can be particularly useful when the base GWAS and target samples are genotyped on a different platform, as then there can be a small fraction of overlapping SNPs. For example, of the 725,459 post-QC SNPs (see Supplementary Material) in the UK Biobank genotype data [10], only 31% (222,956) of those were found in the GIANT Height and Body Mass Index (BMI) GWAS [20,21]. The use of imputed SNPs increases the number of overlapping SNPs to 2,121,036 SNPs. To assess the gain in power when using imputed vs un-imputed data, we performed PRS analyses on height and BMI using UK Biobank genotyped and imputed data, with GWAS summary statistics provided by the GIANT consortium [20,21]. Age, sex, UK Biobank genotyping batch, UK Biobank assessment centre and 40 principle components were first regressed out from the phenotype and the standardized residuals were used instead.

We performed a linear regression using PRSice-2, with the UK Biobank data as target sample using the default parameters. When calculating PRS from the "best-guess" genotype, the "best-guess" genotype is defined as the genotype having an imputation probability of 0.9 or above. If there is no such genotype, then the SNP is considered to be missing for the individual. In addition, for the imputed data, we filtered out SNPs with imputation quality score less than 0.8. With height as the outcome and PRS for height as predictor, we observed an increase in phenotypic variance explained (R^2) of the PRS from 0.145 when using genotyped data to 0.152 when using best-guess imputed genotypes, and 0.153 when using dosage data; likewise, the R^2 for BMI increased from 0.0475 when using genotype data to 0.0529 when using best-guess genotypes, and to 0.0535 when using dosage data. These results exemplify the potential gain in predictive power when using dosage data compared to using genotyped or best-guess genotype data. However, given the modest increases in predictive power, users may wish to perform first-pass analyses on genotyped-only data before application to the more computationally intensive imputed data. A further challenge in exploiting imputed data is that there are numerous imputed formats in use in the field. While it is difficult to support all imputed formats, PRSice-2 adopts a modular approach, which allows simple incorporation of supports for additional data formats (eg. vcf) in the future.

Calculation of Empirical *P*-value

All approaches to PRS calculation involve parameter optimisation in generating the final prediction model, and are thus vulnerable to overfitting [14]. The best strategy to avoid overfitting is to evaluate performance in an independent validation sample, but such a sample is not always available. Alternatively, if the primary aim is to assess evidence for an association to test a

hypothesis, then we can calculate an empirical *P*-value corresponding to the association of the optimized PRS, with the Type 1 error rate controlled [13].

In PRSice-2, to obtain the empirical *P*-value, the target trait values are permuted across the sample of individuals *k* times (default = 10,000) and the PRS analysis is repeated on each set of permuted phenotypes. Thus, on each permutation, the "best-fit PRS" is obtained as that most associated with the target trait across the range of *P*-value thresholds considered, and the empirical *P*-value is calculated as:

$$empirical\ P = \frac{\sum_{n=1}^{N} I(P_n < P_o) + 1}{N + 1} \tag{2}$$

where N is the number of permutations performed, I(.) is the indicator function, which takes a value of 0 if the "best-fit PRS" of permutation *n* is smaller than the observed *P*-value, P_o, and where pseudo-counts of 1 are added to the numerator and denominator to avoid empirical *P*-values of 0 and reflecting (conservatively) counting the observed trait configuration as one potential null permutation [22]. While the empirical *P*-values for association will be controlled for the Type 1 error rate, since the same process of parameter optimisation is performed explicitly under the null hypothesis, the observed phenotypic variance explained, R^2, remains unadjusted and is affected by overfitting. Therefore, it is imperative to perform out-of-sample prediction, or cross-validation, to evaluate the predictive accuracy of PRS when using PRSice-2, and ideally the former given the problems of generalisability observed with PRS [14].

Analysis of PRS strata

While PRS on most complex traits presently have limited power to accurately predict risk at the individual-level, which will remain the case for low-moderate heritability traits irrespective of

GWAS sample sizes, recent studies have demonstrated that individuals at the tails of PRS distribution can have substantially higher disease risk than those of the general population. Thus, these individuals may provide useful subjects for experimental follow-up, while in clinical settings it could be more efficacious to employ different risk management strategies, in terms of screening or interventions, for example, for individuals with extreme PRS [1–3].

We have implemented a strata analysis feature in PRSice-2 to aid the calculation of relative phenotypic risk of individuals between strata. Briefly, the N individuals of the target sample are first aggregated into M different strata based on their PRS. An N x $(M-1)$ design matrix is then generated using dummy coding, such that an individual is coded 1 in the column that corresponds to their PRS stratum and whereby a user-defined stratum is the reference group (or the median stratum by default). A linear regression (for quantitative traits) or logistic regression (for binary traits) will then be performed to estimate the phenotypic difference or relative risk, respectively, of each stratum versus the reference. The set of corresponding beta-coefficients (linear) or the odds ratio (logistic), can then be visualized with the strata plot (Figure 1). This allow users to assess whether individuals in the extreme stratum have a substantially higher phenotypic risk when compared to the reference stratum.

Figure 1

Figure 1 Strata plot generated by PRSice-2. The X-axis shows the range of different quantiles (eg. (80,90] corresponds to those individuals with PRS between the 80%-ile – 90%-ile of the population), and the Y-axis shows the odds ratio (OR) when comparing PRS from different quantiles with the reference quantile (here, (40,60]).

Benchmarking

Here we perform a simulation study to compare the performance of PRSice-2 to alternative polygenic score software lassosum [15] and LDpred [16], in terms of runtime, memory usage and predictive power.

Quantitative traits with heritability (h^2) of 0.2 and 0.6 were simulated with the UK Biobank genotype data (post-QC) as input. Briefly, each quantitative trait was simulated based on the following linear model:

$$Y = X\beta + \varepsilon \tag{3}$$

where X is the unstandardized genotype matrix corresponding to 385,794 individuals (rows) and 560,173 SNP genotypes (columns). The β vector corresponds to the effect size associated with each SNP, with 100, 1k, 10k, 100k and 560,173 (all SNPs) randomly selected to be causal SNPs with effect size $\beta \sim N(0,1)$, β = 0 otherwise, and ε represents the random error, which follows $\varepsilon \sim N\left(0, \sqrt{\frac{var(X\beta)(1-h^2)}{h^2}}\right)$. To control for batch effects and population structure in the genotype data, a regression of batch and 40 PCs against the simulated trait were performed as follows:

$$Y = Batch + 40\ PCs + \varepsilon \tag{4}$$

The standardized residuals were then used as the final simulated trait. Samples of size 50k and 200k individuals were randomly selected as the base sample and used to generate the GWAS summary statistics. 100, 1k, 10k and 100k samples independent from the base were then randomly selected as the target sample. PRS analyses were then performed on these base and target data using the latest version of lassosum (v0.4.4), LDpred (v1.0.6) and PRSice 2 (v2.2.1), on servers equipped with 286 Intel 8168 24 core @ 2.7GHz and 192GB of RAM. Default parameters of each program were used. The runtime and memory usage of each program were measured using the

Linux *time* command and the predictive power of the methods was assessed according to phenotypic variance explained (R^2). The entire process was repeated 10 times to obtain an estimated distribution of runtime, memory usage and predictive power.

Figure 2 shows the runtime and memory usage of PRSice-2, lassosum and LDpred. Based on these simulation results, PRSice-2 is the most efficient software in all settings (Figure 2a), significantly faster than lassosum (P = 1e-58, one sided t-test) and LDpred (P = 2e-90, one sided t-test). Specifically, PRSice-2 can complete the full PRS analysis on 100k samples within 4 minutes (Supplementary Table 1), which is 179x faster than the 10 hours required by lassosum, and 241x faster than the 13 hours 27 minutes required by LDpred. Likewise, PRSice-2 requires significantly less memory (Figure 2b) than lassosum (P = 1e-202, one sided t-test) and LDpred (P = 9e-112, one sided t-test), requiring less than 500MB of memory for 100k samples, as opposed to 11.2 GB required by lassosum and 45.2 GB required by LDpred (Supplementary Table 2). Likewise, PRSice-2 outperforms PRSice-v1.25, requiring 400x less time and 8x less memory for a target sample size of 10k (similar memory for small target samples. See Supplementary Figure 1, Supplementary Tables 1,2 for details). As data size grows, or when more sophisticated PRS analyses are performed at scale [5,23], these gains in computational efficiency could become even more important.

Figure 2a	Figure 2b

Figure 2 Performance of the three PRS software on simulated data. a) Average run time (in minutes) required to complete the entire analysis, across 10 repeats, when applied to different sizes of target sample. b) Average memory (in GB) required for the different software to process the different sizes of target sample.

Figure 3 shows the predictive power of PRSice-2 when compared to lassosum and LDpred for quantitative traits with heritability of 0.2, base sample size of 50k and target sample size of 10k (see Supplementary Figure 2 for comparisons across all settings). Consistent with previous findings [15,24,25], PRSice-2 has comparable predictive power to lassosum and LDpred, typically generating PRS with predictive power higher than that of LDpred but not as high as lassosum. However, these results are inherently dependent on our modelling assumptions. For example, in our simulation, effect sizes and residual effects are assumed to have a Gaussian distribution and all "causal" SNPs are included in the dataset. Thus, we provide these results only as an approximate guide of performance in settings that match our assumptions. We provide our simulation code (https://github.com/choishingwan/PRSice-paper-script) for others to inspect and repeat our analyses.

While PRS generated by PRSice-2 do not appear to fully optimize predictive accuracy, the simple approach and typically fewer SNPs exploited allows for easier interpretation of the results compared to methods that use all SNPs [26]. Moreover, the efficiency and predictive power of PRSice-2 makes it an ideal tool to perform PRS analyses at scale.

Figure 3

Figure 3 Predictive accuracy of the three PRS software for a simulated trait with heritability h2=0.2,target sample size of 10k and base sample size of 50k. The three programs were run using their default parameter settings. The Y-axis represents the trait variance explained (R^2) by the PRS generated from each software, while the X-axis corresponds to the number of causal SNPs for the simulated trait. Full results of the comparison study are shown in Supplementary Figure 2.

Discussion

We have introduced PRSice-2, a software for the automation of polygenic risk score (PRS) analyses applied to large-scale genotype-phenotype data. Our results demonstrate that PRSice-2 is the most efficient among the leading PRS software, outperforming lassosum [15] and LDpred [16].

As data sizes increase and more sophisticated PRS analyses, such as multi-trait or gene-set based PRS analyses, become common, the efficiency advantages of PRSice-2 will become increasingly important.

Over-fitting is a concern for all approaches to PRS analyses [14]. To control for the Type 1 error rate caused by over-fitting when exploiting PRS for hypothesis testing, PRSice-2 implements the calculation of empirical *P*-values.

PRSice-2 implements a standard approach for performing PRS analyses. For PRS analyses performed in family data or across diverse populations, for instance, results should be interpreted carefully [14] and extensions of the standard PRS approach or alternatives may be required [14,27–29] to generate more informative results.

Availability and requirements

**Project Name**	PRSice-2
**Project home page**	http://prsice.info
**Operating systems (pre-compiled versions)**	Linux (64-bit)<br>OS X (64-bit Intel)<br>Windows (64-bit)
**Programming language**	C++, R (version 3.2.3+)
**Other requirements (when recompiling)**	GCC version 4.8+, zlib

**License**	GNU General Public License version 3.0 (GPLv3)
**Any restrictions to use by non-academics**	None
**RRID**	SCR_017057

Availability of Supporting Data

Data further supporting this work and snapshots of the code are available in the *GigaScience* repository, GigaDB [30].

Declarations

Abbreviations

BMI: Body Mass Index; GWAS: Genome Wide Association Study; SNP: Single Nucleotide Polymorphism; PRS: Polygenic Risk Score

Competing Interests

The authors declare that they have no competing interests

Funding

Medical Research Council FundRef identification ID: http://dx.doi.org/10.13039/501100000265 MR/N015746/1 to Paul F. O'Reilly

Authors' contributions

Conceptualization, SWC and PFO; Methodology, SWC and PFO; Investigation, SWC; Software, SWC; Supervision, PFO; Funding Acquisition, PFO; Writing - Original Draft, SWC; Writing - Review & Editing, SWC and PFO.

Acknowledgements

We thank the participants in the UK Biobank and the scientists involved in the construction of this resource. This research has been conducted using the UK Biobank Resource under application

18177 (Dr O'Reilly). We thank Hei Man Wu for providing critical feedback regarding this manuscript and for test running the software. We thank Jonathan Coleman and Kylie Glanville for the management of the UK Biobank resource at King's College London, and we thank Jack Euesden for his work on PRSice, which forms the basis of the current software. We thank Christopher Hübel, Eva Krapohl, Kirstin Purves, Jessye Maxwell, Saskia Hagenaars and Yunfeng Ruan for their help in test running the software. This work was supported in part through the computational resources and staff expertise provided by the Department of Scientific Computing at the Icahn School of Medicine at Mount Sinai. PFO receives funding from the UK Medical Research Council (MR/N015746/1). SWC is funded from the UK Medical Research Council (MR/N015746/1). This report represents independent research (part)-funded by the National Institute for Health Research (NIHR) Biomedical Research Centre at South London and Maudsley NHS Foundation Trust and King's College London. The views expressed are those of the authors and not necessarily those of the NHS, the NIHR, or the Department of Health.

References

1. Mavaddat N, Pharoah PDP, Michailidou K, Tyrer J, Brook MN, Bolla MK, et al. Prediction of Breast Cancer Risk Based on Profiling With Common Genetic Variants. JNCI J Natl Cancer Inst [Internet]. 2015 [cited 2017 Jun 13];107. Available from: https://academic.oup.com/jnci/article/107/5/djv036/891009/Prediction-of-Breast-Cancer-Risk-Based-on

2. Kuchenbaecker KB, McGuffog L, Barrowdale D, Lee A, Soucy P, Healey S, et al. Evaluation of Polygenic Risk Scores for Breast and Ovarian Cancer Risk Prediction in BRCA1 and BRCA2 Mutation Carriers. JNCI J Natl Cancer Inst [Internet]. 2017 [cited 2018 Sep 26];109. Available from: https://www.ncbi.nlm.nih.gov/pmc/articles/PMC5408990/

3. Natarajan P, Young R, Stitziel NO, Padmanabhan S, Baber U, Mehran R, et al. Polygenic Risk Score Identifies Subgroup with Higher Burden of Atherosclerosis and Greater Relative Benefit from Statin Therapy in the Primary Prevention Setting. Circulation. 2017;CIRCULATIONAHA.116.024436.

4. Udler MS, Kim J, Grotthuss M von, Bonas-Guarch S, Mercader JM, Cole JB, et al. Clustering of Type 2 Diabetes Genetic Loci by Multi-Trait Associations Identifies Disease Mechanisms and Subtypes. bioRxiv. 2018;319509.

5. Krapohl E, Euesden J, Zabaneh D, Pingault J-B, Rimfeld K, von Stumm S, et al. Phenome-wide analysis of genome-wide polygenic scores. Mol Psychiatry. 2016;21:1188–93.

6. Krapohl E, Patel H, Newhouse S, Curtis CJ, Stumm S von, Dale PS, et al. Multi-polygenic score approach to trait prediction. Mol Psychiatry. 2018;23:1368–74.

7. Selzam S, Krapohl E, von Stumm S, O'Reilly PF, Rimfeld K, Kovas Y, et al. Predicting educational achievement from DNA. Mol Psychiatry. 2017;22:267–72.

8. Selzam S, Dale PS, Wagner RK, DeFries JC, Cederlöf M, O'Reilly PF, et al. Genome-Wide Polygenic Scores Predict Reading Performance Throughout the School Years. Sci Stud Read. 2017;21:334–49.

9. Du Rietz E, Coleman J, Glanville K, Choi SW, O'Reilly PF, Kuntsi J. Association of Polygenic Risk for Attention-Deficit/Hyperactivity Disorder With Co-occurring Traits and Disorders. Biol Psychiatry Cogn Neurosci Neuroimaging. 2018;3:635–43.

10. Sudlow C, Gallacher J, Allen N, Beral V, Burton P, Danesh J, et al. UK Biobank: An Open Access Resource for Identifying the Causes of a Wide Range of Complex Diseases of Middle and Old Age. PLOS Med. 2015;12:e1001779.

11. Danciu I, Cowan JD, Basford M, Wang X, Saip A, Osgood S, et al. Secondary use of clinical data: The Vanderbilt approach. J Biomed Inform. 2014;52:28–35.

12. Kaiser J. NIH's 1-million-volunteer precision medicine study announces first pilot projects. Science [Internet]. 2016 [cited 2018 Nov 15]; Available from: https://www.sciencemag.org/news/2016/02/nih-s-1-million-volunteer-precision-medicine-study-announces-first-pilot-projects

13. Euesden J, Lewis CM, O'Reilly PF. PRSice: Polygenic Risk Score software. Bioinformatics. 2015;31:1466–8.

14. Choi SW, Mak TSH, O'Reilly P. A guide to performing Polygenic Risk Score analyses. bioRxiv. 2018;416545.

15. Mak TSH, Porsch RM, Choi SW, Zhou X, Sham PC. Polygenic scores via penalized regression on summary statistics. Genet Epidemiol. 2017;41:469–80.

16. Vilhjálmsson BJ, Yang J, Finucane HK, Gusev A, Lindström S, Ripke S, et al. Modeling Linkage Disequilibrium Increases Accuracy of Polygenic Risk Scores. Am J Hum Genet. 2015;97:576–92.

17. Chang CC, Chow CC, Tellier LC, Vattikuti S, Purcell SM, Lee JJ. Second-generation PLINK: rising to the challenge of larger and richer datasets. GigaScience. 2015;4:7.

18. Wray NR, Lee SH, Mehta D, Vinkhuyzen AAE, Dudbridge F, Middeldorp CM. Research review: Polygenic methods and their application to psychiatric traits. J Child Psychol Psychiatry. 2014;55:1068–87.

19. Li Y, Willer C, Sanna S, Abecasis G. Genotype Imputation. Annu Rev Genomics Hum Genet. 2009;10:387–406.

20. Wood AR, Esko T, Yang J, Vedantam S, Pers TH, Gustafsson S, et al. Defining the role of common variation in the genomic and biological architecture of adult human height. Nat Genet. 2014;46:1173–86.

21. Locke AE, Kahali B, Berndt SI, Justice AE, Pers TH, Day FR, et al. Genetic studies of body mass index yield new insights for obesity biology. Nature. 2015;518:197–206.

22. North BV, Curtis D, Sham PC. A Note on the Calculation of Empirical P Values from Monte Carlo Procedures. Am J Hum Genet. 2002;71:439–41.

23. Hagenaars SP, Harris SE, Davies G, Hill WD, Liewald DCM, Ritchie SJ, et al. Shared genetic aetiology between cognitive functions and physical and mental health in UK Biobank (N=112 151) and 24 GWAS consortia. Mol Psychiatry. 2016;21:1624–32.

24. Allegrini A, Selzam S, Rimfeld K, Stumm S von, Pingault J-B, Plomin R. Genomic prediction of cognitive traits in childhood and adolescence. bioRxiv. 2018;418210.

25. Ge T, Chen C-Y, Ni Y, Feng Y-CA, Smoller JW. Polygenic Prediction via Bayesian Regression and Continuous Shrinkage Priors. bioRxiv. 2018;416859.

26. Janssens ACJW, Joyner MJ. Polygenic Risk Scores That Predict Common Diseases Using Millions of Single Nucleotide Polymorphisms: Is More, Better? Clin Chem. 2019;clinchem.2018.296103.

27. Duncan L, Shen H, Gelaye B, Ressler K, Feldman M, Peterson R, et al. Analysis of Polygenic Score Usage and Performance across Diverse Human Populations. bioRxiv. 2018;398396.

28. Márquez- Luna C, Loh P-R, Price AL. Multiethnic polygenic risk scores improve risk prediction in diverse populations. Genet Epidemiol. 2017;41:811–23.

29. Martin AR, Gignoux CR, Walters RK, Wojcik GL, Neale BM, Gravel S, et al. Human Demographic History Impacts Genetic Risk Prediction across Diverse Populations. Am J Hum Genet. 2017;100:635–49.

30. Choi SW; O'Reilly PF: Supporting data for "PRSice-2: Polygenic Risk Score Software for Large-Scale Data" *GigaScience* Database. 2019. http://dx.doi.org/10.5524/100591.

Figure 2a

Click here to access/download;Figure;Figure 2a.png

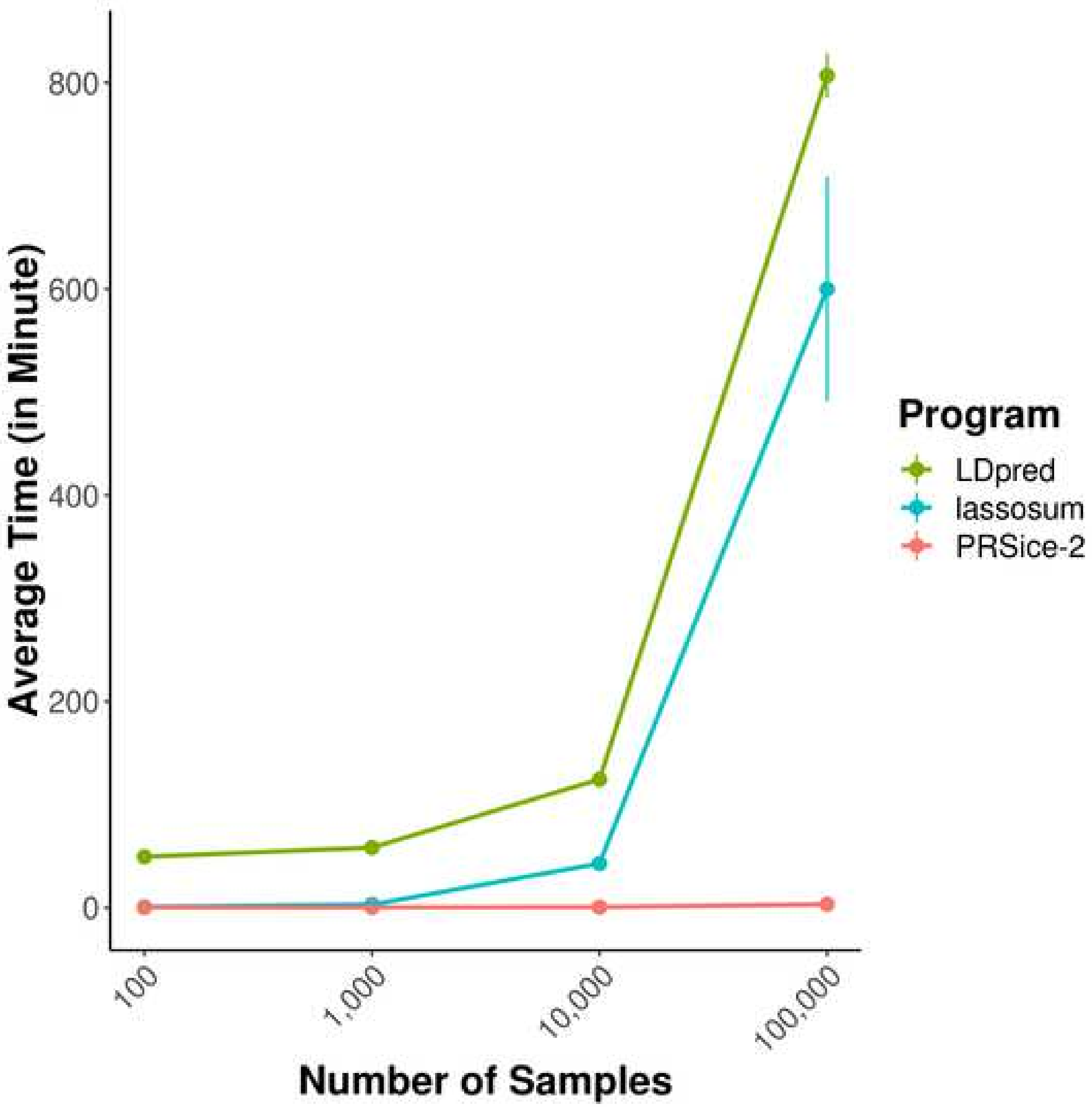

Figure 2b

Click here to access/download;Figure;Figure 2b.png

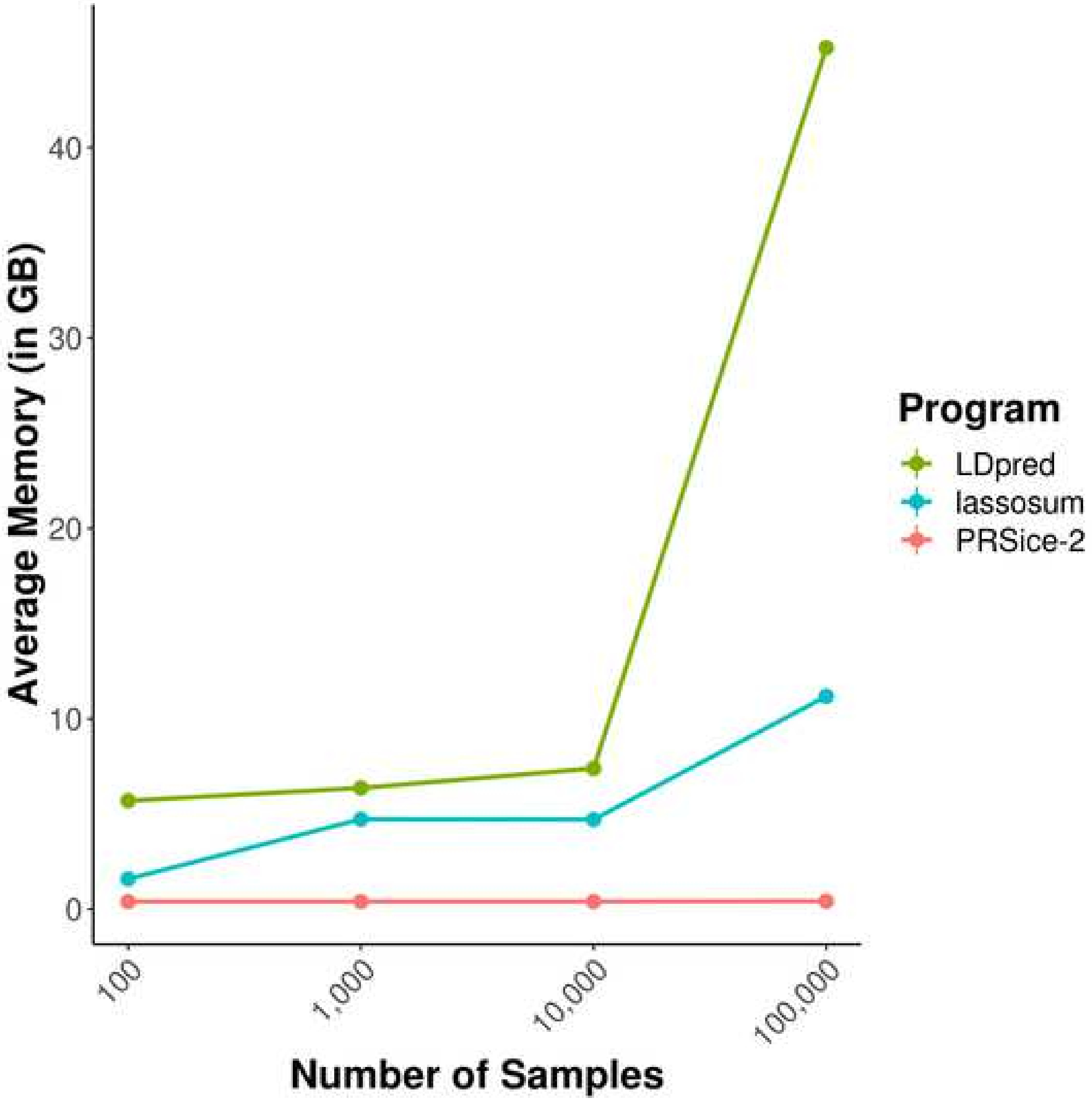

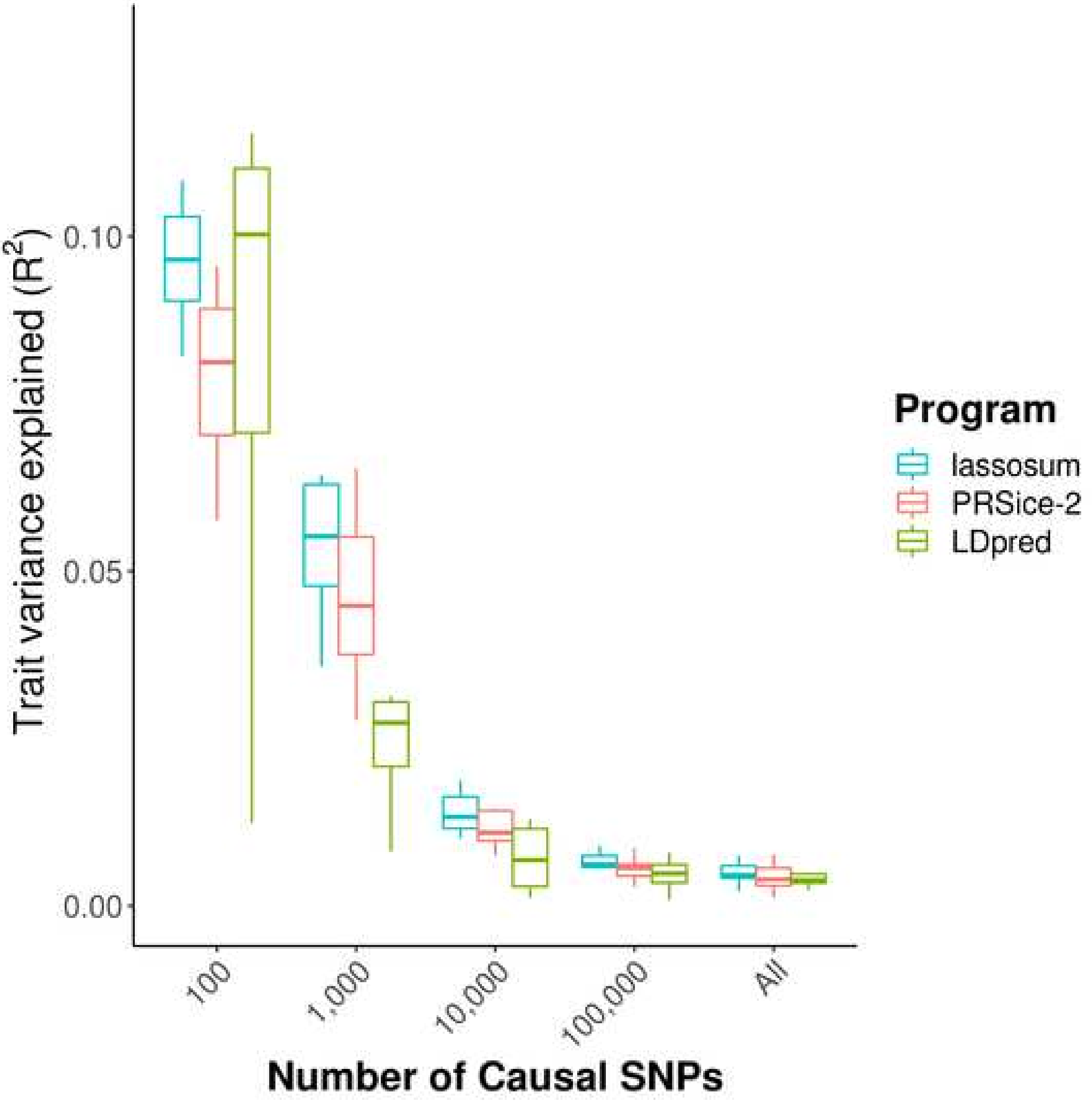

Trait variance explained (R^2)
0.10
0.05
0.00
100
1,000
10,000
100,000
All
Number of Causal SNPs
Program
lassosum
PRSice-2
LDpred

Click here to access/download
Supplementary Material
20190604-PRSice2 Supplementary.docx

Personal Cover

Click here to access/download;Personal Cover;20190611-CoverLetter_PRSice2.docx

MRC Social, Genetic &
Developmental Psychiatry Centre
Director **Francesca Happé**

16 De Crespigny Park
Denmark Hill
London SE5 8AF

Dr Shing Wan Choi
Email: shing_wan.choi@kcl.ac.uk
Telephone: +44 (0)7729246486

PRSice-2: Polygenic Risk Score Analysis Software for Biobank-Scale Data *(For submission as a Technical Note)*

11th June 2019

Dear Editor,

Thank you for your patience. After the previous exchange, we agreed with reviewer 2 that the poor performance of LDpred seemed peculiar. As a result of that, we contacted the first author of LDpred, Dr Bjarni Vilhjalmsson. He informed us that LDpred can become sensitive to small deviations in LD estimates when there are large sample sizes in application to a trait with high heritability. We also noted that there is a new version of LDpred (v1.0.6) now available. Repeating our analyses using a smaller base sample size of 50000, and using the latest version of LDpred, we noted that the performance of LDpred substantially improved. As a result of this, we repeated our entire analyses using the latest versions of PRSice-2 and LDpred and have updated our results accordingly. The overall results remain qualitatively unchanged: PRSice-2 is still markedly the fastest PRS program (more so than previously) and it has comparable power to lassosum and LDpred, with predictive power higher than LDpred and lower than lassosum. Overall, we believe that our final manuscript is much improved and also up-to-date to the very latest versions of all programs.

Thank you very much for your assistance and patience with this, and for selecting our manuscript for publication in Gigascience. In a minor point, we note that we have changed the title to state ‘biobank-scale data’ rather than ‘large-scale data’ as we felt that this was more appropriate (and also engaging) in the context, but we understand if it is too late for such a change.

Shing Wan Choi (cc’ed Paul F. O’Reilly)
