# Supplementary material for: PRSice-2: Polygenic Risk Score software for biobank-scale data
Source: Gigascience. 2019 Jul 15;8(7):giz082. doi: 10.1093/gigascience/giz082 (PMC6629542; doi:10.1093/gigascience/giz082)
Supplement: giz082_Supplemental_File [file giz082_supplemental_file.docx]

# Supplementary Method and Tables

## UK Biobank Samples and Quality Control

UK Biobank is a large national health resources consists of 502,655 people aged between 40-69 years in 2006-2010 across the United Kingdom. Genotyping was performed using the Affymetrix UK BiLEVE Axiom array and the Affymetrix UK Biobank Axiom® array [1]. Standard quality controls were performed to filter variants and participants: remove SNPs with minor allele frequency less than 0.01, genotype missingness higher than 0.02, individual missingness higher than 0.02, SNPs with Hardy-Weinberg Equilibrium P-value less than 1x10^-8^ and samples with mismatch sex information. A list of individuals of European ancestry were created using 4-means clustering on the first two principal components provided by the UK Biobank, samples from non-European ancestry were then removed. Related samples were excluded based on the relatedness kinship (KING) estimates, where one individual from each pair with up to 3rd-degree relationships (KING r^2^ > 0.044) [2] were removed using a “greedy” algorithm to minimize exclusion [3]. A total of 385,794 samples and 560,173 SNPs are remained after quality control. For imputed data, SNPs with low imputation quality score (INFO < 0.8) were also filtered, result in a total of 10,926,551 imputed SNPs

| a)  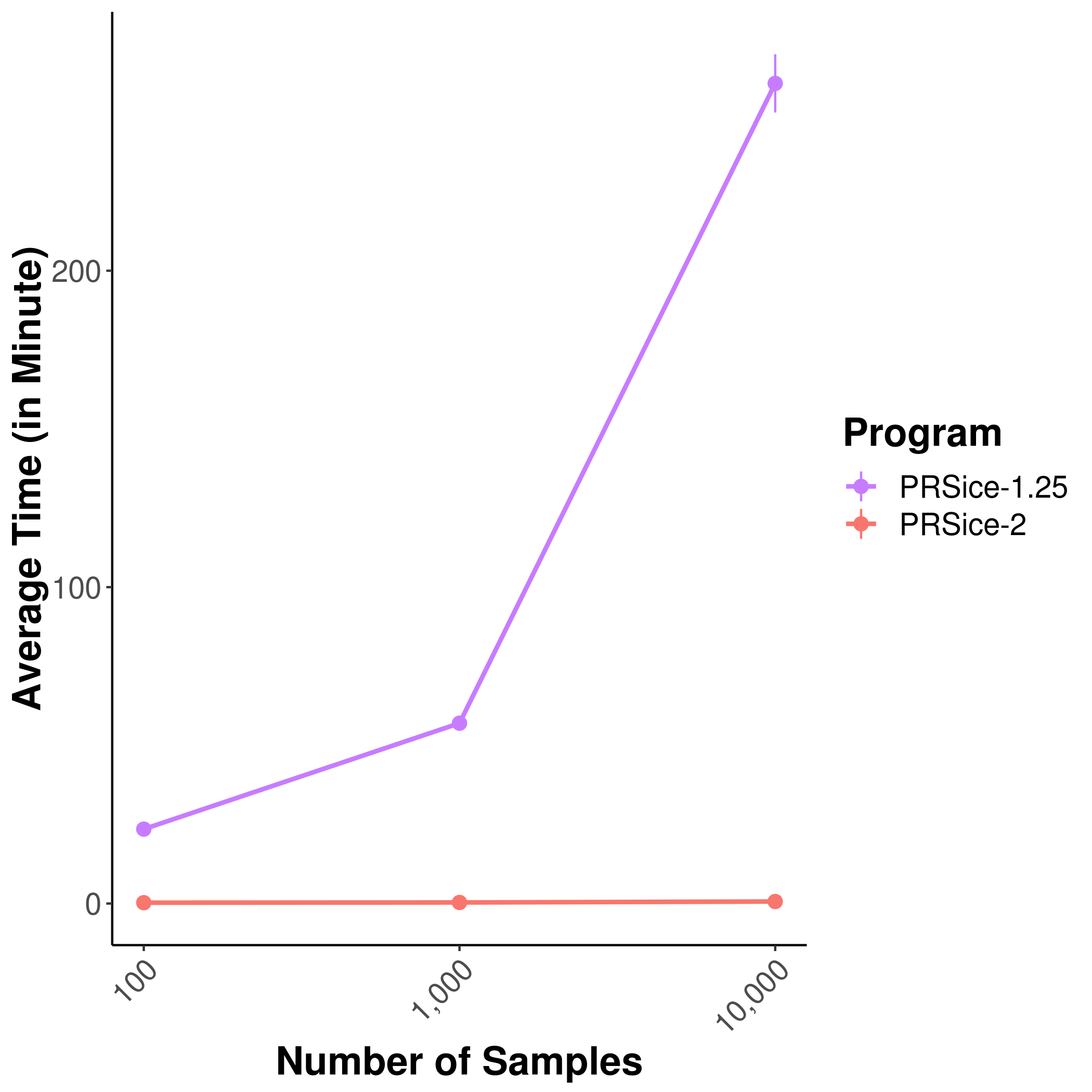 | b)  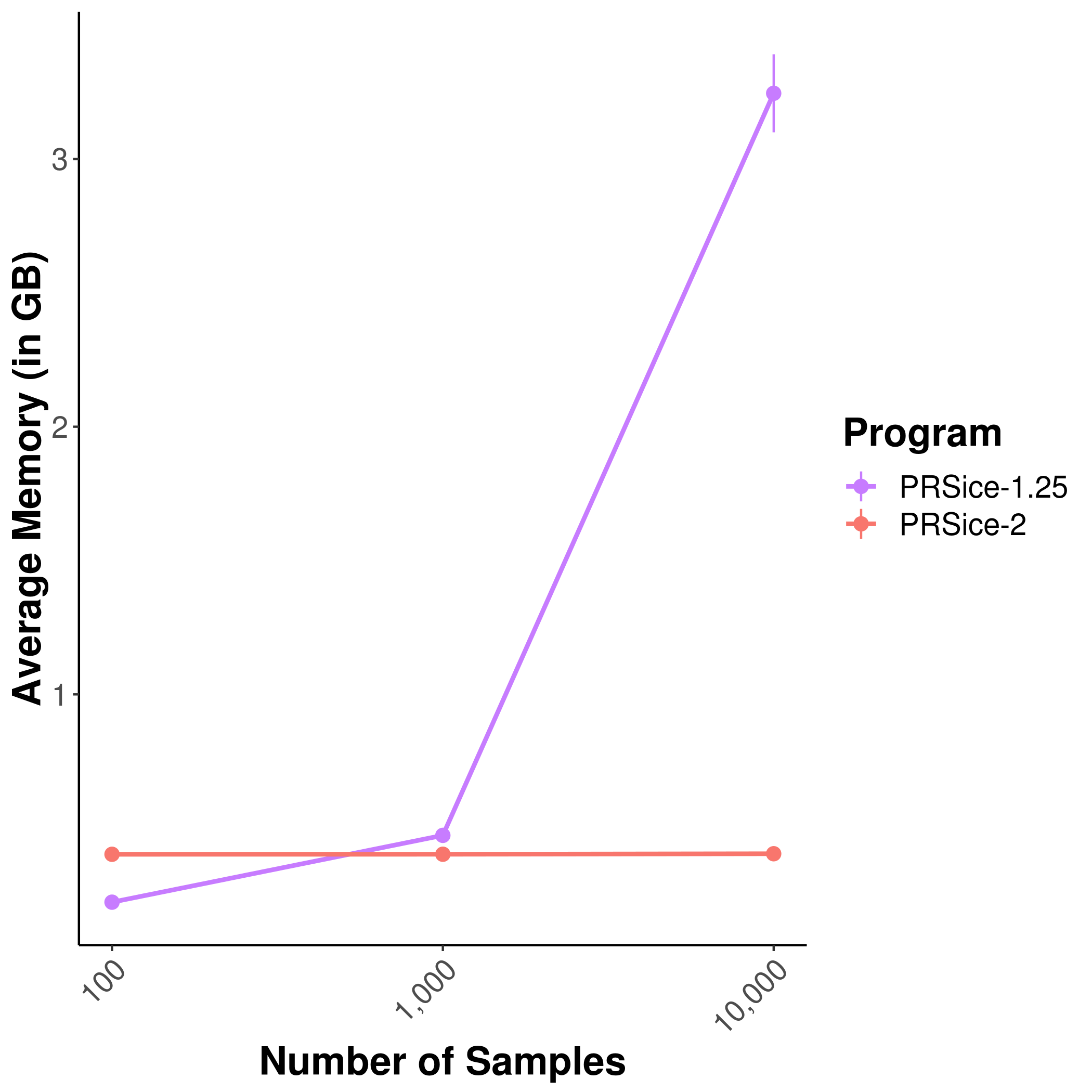 |
| --- | --- |

Supplementary Figure 1 Performance of PRSice-2 compared to PRSice-1.25. a) Average run time (in minutes) required to complete the entire analysis, across 10 repeats, when applied to different sizes of target sample. b) Average memory (in GB) required to process the different sizes of target sample.

| Program | Sample Size | | | |
| --- | --- | --- | --- | --- |
|  | 100 | 1000 | 10000 | 100000 |
| PRSice-2 | 0.266  (0.000608) | 0.339  (0.000703) | 0.635  (0.00151) | 3.34  (0.00655) |
| lassosum | 0.822  (0.0148) | 3.09  (0.0244) | 42.9  (0.416) | 600  (7.70) |
| LDpred | 49.5  (0.121) | 58.4  (0.177) | 125  (0.189) | 807  (1.53) |
| PRSice | 23.5  (0.0776) | 57.0  (0.233) | 259  (0.920) | - |

Supplementary Table 1 Average run time (in minutes) for each program over the 10 iterations, across different heritability and number of causal SNPs. Standard error of run time is in brackets. Due to the large number of intermediate files generated by PRSice-1.25 and the excessive runtime required, we did not test the runtime of PRSice-1.25 with 100k target samples.

| Program | Sample Size | | | |
| --- | --- | --- | --- | --- |
|  | 100 | 1000 | 10000 | 100000 |
| PRSice-2 | 0.402  (5.64e-6) | 0.403  (5.31e-6) | 0.405  (5.86e-6) | 0.422  (2.32e-5) |
| lassosum | 1.59  (0. 00324) | 4.73  (0. 000844) | 4.71  (0.00221) | 11.2  (0. 00731) |
| LDpred | 5.70  (0.000746) | 6.36  (0.000182) | 7.39  (0.000213) | 45.2  (0.00111) |
| PRSice | 0.224 (0.000202) | 0.473  (0.00108) | 3.25  (0.0146) | - |

Supplementary Table 2 Average memory usage (in GB) for each program over the 10 iterations, across different heritability and number of causal SNPs. Standard error of memory usage is in brackets. Due to the large number of intermediate files generated by PRSice-1.25 and the excessive runtime required, we did not test the memory use of PRSice-1.25 with 100k target samples.


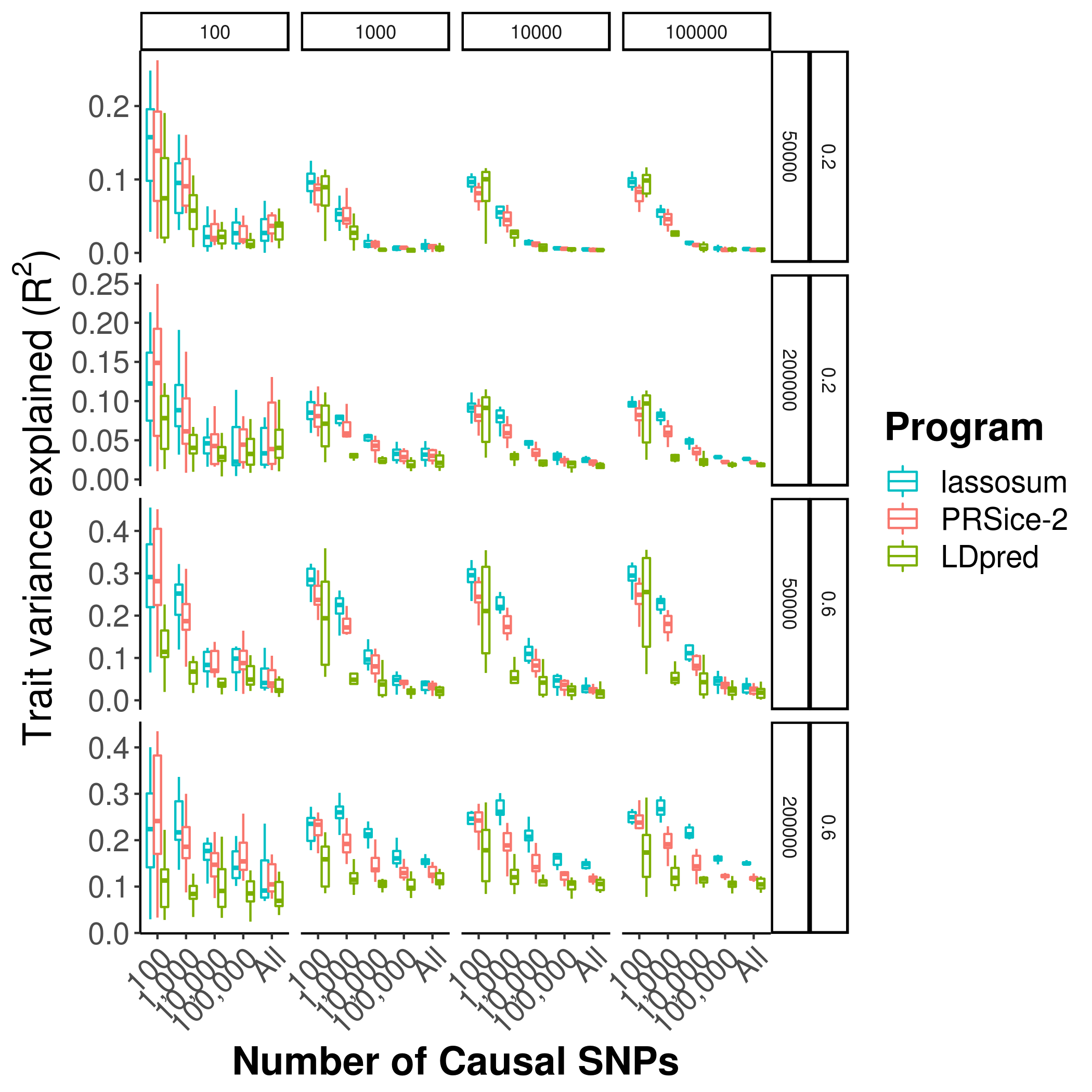


Supplementary Figure 2 Predictive accuracy of the three PRS software across all simulated scenarios using the default parameters. The Y-axis represents the trait variance explained (R^2^) by the PRS generated from each software, while the X-axis corresponds to the number of causal SNPs for the simulated trait. The right side of the graph shows the number of base samples included in the simulation and heritability of the simulated trait while the top of the graph shows the number of target samples included in the simulation.
