# Supplementary material for: PRSice-2: Polygenic Risk Score software for biobank-scale data
Source: Gigascience. 2019 Jul 15;8(7):giz082. doi: 10.1093/gigascience/giz082 (PMC6629542; doi:10.1093/gigascience/giz082)
Supplement: giz082_Response_to_Reviewer_Comments_Original_Submission [file giz082_response_to_reviewer_comments_original_submission.pdf]

Author's Response To Reviewer Comments

Close

Reviewer #1: Choi et al. have proposed a new extension of their PRSice method. The new method, PRSice-2, main advantage is speed as most of the code is written in C++ and PRSice-2 avoid creating intermediate files.

Major Comments:

1. The authors claim that their method is faster and more memory efficient than LDpred and lassosum. However, the authors need to compare these methods in case of prediction accuracy as well.

>> Thank you for your suggestion, which we think has now made our Technical Note more comprehensive. We have now included a full simulation analysis investigating the predictive accuracy of PRSice-2 compared to LDpred and lassosum (see Figure 3 and Supplementary Figure 2).

2. I like to see experiments where the authors compare PRSice-2 with PRSice performance.

>> We have now performed a comparison between PRSice-2 and PRSice-v1.25, both in terms of speed and memory (predictive accuracy is the same given the same underlying approach). Results can be found in Supplementary Figure 1, Supplementary Table 1 and Supplementary Table 2

Minor Comments:

The authors need to comment regarding the case where we have multiple populations in a study. For example Luna et al. Genetic epidemiology 2017 work discuss how to solve this problem.

The authors need to mention some of their method limitations in the discussion section.

>> Thank you for your comment. We agree that differences in allele frequencies, linkage disequilibrium and factors such as genetic drift and natural selection between populations can reduce the generalisability of PRS analyses across populations and produce misleading results, as suggested by Martin et. al. (2017) and as described in our 'Guide to performing polygenic risk score analyses' (Choi, Mak, O'Reilly. 2018. bioRxiv). We have now described this issue in our discussion, citing Duncan et al, Luna et al, Martin et al and Choi et al, and we caution users to take extra care when performing cross-population and family-wise PRS analyses.

Reviewer #2: This article reports the release of a new version of the PRSice software for polygenic score calculation. The new version of the software boasts speed enhancements that make it appealing for applications in the growing number of ultra-large genetically-informed datasets including the UK Biobank, 23andMe and others. Also important are features allowing for polygenic score computation from imputed genotype datasets in which genotypes are represented as a probabilities rather than discrete allele counts.

The data on speed are compelling. This alone is a good argument for why PRSice v1 users should upgrade to v2. But I found the article thinner on two other key questions central to addressing whether those not already using PRSice v2 should take up PRSice v2:
(1) Does the polygenic scoring method implemented within PRSice2 (additive combination of SNPs with/without LD clumping) deliver comparably predictive scores to other software, e.g. the LDPred and lassosum softwares?

>> Thank you for your comment and we agree that this is an important question. To address this, we have now performed a comprehensive simulation analysis to demonstrate the predictive power of PRSice-2 Vs LDpred and lassosum (see Figure 3 and Supplementary Figure 2).

(2) What is the value added of being able to accommodate imputed genotype probabilities rather than

relying exclusively on discrete allele count data?

>> We thank the reviewer for this comment. We have now also performed an analysis to compare the predictive power of PRS constructed from genotyped data, or from imputed data either in terms of best-guess genotypes or dosage values. Briefly, the R2 for the Height PRS increased from 0.145 when using genotyped data to 0.152 when using best-guess imputed genotypes, and to 0.153 when using dosage data; likewise the R2 for BMI increased from 0.0475 when using genotype data to 0.0529 when using best-guess genotypes, and to 0.0535 when using dosage data.

I would suggest the following revisions:

Re PRSice2 vs. Alternative Softwares: The authors assert that the method of polygenic score calculation implemented within PRSice2 generates scores that are comparably predictive to two other methodologies, LDPred and LassoSum. It is my understanding that these methods were developed and are in use precisely because they outperform the method implemented in PRSice in terms of the prediction R-squared for the target phenotype. It would improve the article if the authors could provide some empirical evidence for the claim that their software delivers polygenic scores of comparable accuracy to other methods. For example, comparison of PRSice2 scores to scores generated from LDPred and lassosum for a set of traits would be helpful. I like the choices of height and BMI. But it might also be sensible to consider a trait for which existing GWAS are smaller/ polygenic predictions are less accurate, e.g. depression.

>> Please see above response

Re Imputed Genotype Probabilities vs. Allele Counts: The authors helpfully report that PRSice2 scores computed with imputed data can improve prediction accuracy by about 1 percentage point for height and BMI as compared to scores computed with genotyped-only data. It would be helpful to add an element to this analysis. As I understand it, the authors are comparing a genotyped-SNP-only polygenic score computed from allele counts to an imputed-SNP polygenic score computed from genotype probabilities. But these are not the only two possibilities. In much polygenic score analysis, imputed SNP probabilities are converted to discrete genotypes using a threshold (e.g. probability=0.9) to determine whether a given genotype can be assigned to the SNP. Since this is common practice in the field, it seems to me that it would be helpful to include this approach in the comparison.

>> Please see above response

Finally, I have one small quibble about language:

In the introduction, the authors assert that polygenic scores have proven clinical utility. This is a bit of an overstatement. I think we can say that "provocative new data suggest the potential for polygenic scores to be useful in clinical settings" or something similar. The recent papers referenced by the authors are compelling. But the term clinical utility has a specific meaning - that application of a tool improves patient outcomes (e.g. see Torkamani et al. 2018 Nat Rev Genet). We are a long way off from that. Instead, the evidence we have supports an argument for the clinical validity of extreme polygenic-scores values for assessing disease risk.

>> We thank the reviewer for highlighting this and we entirely agree, that as worded, this could have led readers to a conclusion that we do not agree with ourselves (ie. we also believe that PRS are a long way off clinical utility at the individual-level). We have now changed the introduction as follows (note mention of 'stratified medicine' in the revised version, as opposed to personalized medicine):

"Polygenic Risk Score (PRS) analyses are beginning to play a critical role in biomedical research, being already sufficiently powered to provide scientific insights and with the potential to contribute to stratified medicine in the future [1-9]."

Close
