# Supplementary material for: PRSice-2: Polygenic Risk Score software for biobank-scale data
Source: Gigascience. 2019 Jul 15;8(7):giz082. doi: 10.1093/gigascience/giz082 (PMC6629542; doi:10.1093/gigascience/giz082)
Supplement: giz082_Response_to_Reviewer_Comments_Revision_1 [file giz082_response_to_reviewer_comments_revision_1.pdf]

Author's Response To Reviewer Comments

Close

We contacted the first author of LDpred, Dr Bjarni Vilhjalmsson. He informed us that LDpred can become sensitive to small deviations in LD estimates when there are large sample sizes in application to a trait with high heritability. We also noted that there is a new version of LDpred (v1.0.6) now available. Repeating our analyses using a smaller base sample size of 50000, and using the latest version of LDpred, we noted that the performance of LDpred substantially improved. As a result of this, we repeated our entire analyses using the latest versions of PRSice-2 and LDpred and have updated our results accordingly. The overall results remain qualitatively unchanged: PRSice-2 is still markedly the fastest PRS program (more so than previously) and it has comparable power to lassosum and LDpred, with predictive power higher than LDpred and lower than lassosum.

Close
