# Supplementary material for: PRSice-2: Polygenic Risk Score software for biobank-scale data
Source: Gigascience. 2019 Jul 15;8(7):giz082. doi: 10.1093/gigascience/giz082 (PMC6629542; doi:10.1093/gigascience/giz082)
Supplement: giz082_Reviewer_2_Report_Original_Submission — Daniel Belsky -- 1/15/2019 Reviewed [file giz082_reviewer_2_report_original_submission.pdf]

Reviewer Report

Title: PRSice-2: Polygenic Risk Score software for biobank-scale data

Version: Original Submission **Date:** 1/15/2019

Reviewer name: Daniel Belsky

Reviewer Comments to Author:

This article reports the release of a new version of the PRSice software for polygenic score calculation. The new version of the software boasts speed enhancements that make it appealing for applications in the growing number of ultra-large genetically-informed datasets including the UK Biobank, 23andMe and others. Also important are features allowing for polygenic score computation from imputed genotype datasets in which genotypes are represented as a probabilities rather than discrete allele counts.

The data on speed are compelling. This alone is a good argument for why PRSice v1 users should upgrade to v2. But I found the article thinner on two other key questions central to addressing whether those not already using PRSice v2 should take up PRSice v2: (1) Does the polygenic scoring method implemented within PRSice2 (additive combination of SNPs with/without LD clumping) deliver comparably predictive scores to other software, e.g. the LDPred and lassosum softwares? (2) What is the value added of being able to accommodate imputed genotype probabilities rather than relying exclusively on discrete allele count data?

I would suggest the following revisions:

Re PRSice2 vs. Alternative Softwares: The authors assert that the method of polygenic score calculation implemented within PRSice2 generates scores that are comparably predictive to two other methodologies, LDPred and LassoSum. It is my understanding that these methods were developed and are in use precisely because they outperform the method implemented in PRSice in terms of the prediction R-squared for the target phenotype. It would improve the article if the authors could provide some empirical evidence for the claim that their software delivers polygenic scores of comparable accuracy to other methods. For example, comparison of PRSice2 scores to scores generated from LDPred and lassosum for a set of traits would be helpful. I like the choices of height and BMI. But it might also be sensible to consider a trait for which existing GWAS are smaller/ polygenic predictions are less accurate, e.g. depression.

Re Imputed Genotype Probabilities vs. Allele Counts: The authors helpfully report that PRSice2 scores computed with imputed data can improve prediction accuracy by about 1 percentage point for height and BMI as compared to scores computed with genotyped-only data. It would be helpful to add an element to this analysis. As I understand it, the authors are comparing a genotyped-SNP-only polygenic score computed from allele counts to an imputed-SNP polygenic score computed from genotype probabilities. But these are not the only two possibilities. In much polygenic score analysis, imputed SNP probabilities are converted to discrete genotypes using a threshold (e.g. probability=0.9) to determine whether a given genotype can be assigned to the SNP. Since this is common practice in the field, it seems to me that it would be helpful to include this approach in the comparison.

Finally, I have one small quibble about language:
In the introduction, the authors assert that polygenic scores have proven clinical utility. This is a bit of an overstatement. I think we can say that "provocative new data suggest the potential for polygenic scores to be useful in clinical settings" or something similar. The recent papers referenced by the authors are compelling. But the term clinical utility has a specific meaning - that application of a tool improves patient outcomes (e.g. see Torkamani et al. 2018 Nat Rev Genet). We are a long way off from that. Instead, the evidence we have supports an argument for the clinical validity of extreme polygenic-scores values for assessing disease risk.

Level of Interest

Please indicate how interesting you found the manuscript: Choose an item.

Quality of Written English

Please indicate the quality of language in the manuscript: Choose an item.

Declaration of Competing Interests

Please complete a declaration of competing interests, considering the following questions:

- Have you in the past five years received reimbursements, fees, funding, or salary from an organisation that may in any way gain or lose financially from the publication of this manuscript, either now or in the future?
- Do you hold any stocks or shares in an organisation that may in any way gain or lose financially from the publication of this manuscript, either now or in the future?
- Do you hold or are you currently applying for any patents relating to the content of the manuscript?
- Have you received reimbursements, fees, funding, or salary from an organization that holds or has applied for patents relating to the content of the manuscript?
- Do you have any other financial competing interests?
- Do you have any non-financial competing interests in relation to this paper?

If you can answer no to all of the above, write 'I declare that I have no competing interests' below. If your reply is yes to any, please give details below.

I declare that I have no competing interests

I agree to the open peer review policy of the journal. I understand that my name will be included on my report to the authors and, if the manuscript is accepted for publication, my named report including any attachments I upload will be posted on the website along with the authors' responses. I agree for my report to be made available under an Open Access Creative Commons CC-BY license

(http://creativecommons.org/licenses/by/4.0/). I understand that any comments which I do not wish to be included in my named report can be included as confidential comments to the editors, which will not be published.

I agree to the open peer review policy of the journal

To further support our reviewers, we have joined with Publons, where you can gain additional credit to further highlight your hard work (see: https://publons.com/journal/530/gigascience). On publication of this paper, your review will be automatically added to Publons, you can then choose whether or not to claim your Publons credit. I understand this statement.

Yes Choose an item.
